# Agricultural sustainability monitoring in arid regions using hybrid deep learning and Landsat 8 imagery in Najran City, Saudi Arabia

**DOI:** 10.1038/s41598-026-58271-x

**Published:** 2026-06-18

**Authors:** Yousef Asiri, Eman A. Alshari, Aisha M. Mashraqi, Ebrahim Mohammed Senan, Hanan T. Halawani, Bandar Hussein Edah Althagafi

**Affiliations:** 1 Department of Computer Science, College of Computer Science and Information Systems, Najran University, Najran, 61441 Saudi Arabia; 2 Science and Engineering Research Center, Najran University, Najran, Saudi Arabia; 3 Department of Artificial Intelligence, Faculty of Computer Science and Information Technology, Al-Razi University, Sana’a, Yemen; 4 Department of Computer Science, Faculty of Computer Science & Informatics, Thamar University, Dhamar, Yemen; 5 Department of Computer Science, Faculty of Applied Sciences, Hajjah University, Hajjah, Yemen; 6 Technical College of Telecommunications and Information, Jeddah University, Jeddah, Saudi Arabia

**Keywords:** Deep learning, Hybrid System, DenseNet121, Najran City, XGBoost, DT, Remote Sensing, Engineering, Environmental sciences, Mathematics and computing

## Abstract

Accurate monitoring of agricultural land is a cornerstone of sustainable land management, particularly in arid regions like Saudi Arabia, where water resources are scarce. Traditional Land Use Land Cover (LULC) classification methods, dependent on manually engineered features, often lack robustness across diverse environmental conditions. While deep learning models like Convolutional Neural Networks (CNNs) automate feature extraction and enhance generalization, their computational complexity can be prohibitive. This research investigates a hybrid methodology to optimize this balance, integrating the powerful feature learning of DenseNet121 with the computational efficiency of advanced machine learning classifiers, specifically Decision Trees (DT) and XGBoost. The objective was to develop a precise and efficient tool for mapping key land covers—especially agricultural areas—in Najran City using 2020 Landsat 8 imagery. The proposed framework extracts complementary spatial and spectral features, which are then classified. Experimental results demonstrated that the DenseNet121-XGBoost hybrid model achieved superior performance, with an overall accuracy of 98.82% and a Kappa coefficient of 0.9638, significantly outperforming the standalone CNN. This study confirms the efficacy of hybrid deep learning for reliable agricultural land monitoring, providing a valuable decision-support tool for promoting sustainable practices in arid environments.

## Introduction

In recent years, cities’ rapid urbanization and development have led to significant changes in the earth’s landscape. Monitoring and classifying these changes are crucial for various applications, including urban planning, environmental management, and disaster preparedness^1^. The city of Najran, characterized by its unique geographical and ecological conditions, has undergone substantial transformations due to natural and anthropogenic factor. Consequently, the accurate classification of earth changes in Najran City holds significant importance^2^. LULC classification is pivotal in understanding and managing the earth’s surface dynamics. Accurate classification of land areas into various categories, forests, urban areas, water bodies, and agricultural regions, is crucial for environmental monitoring, urban planning, natural resource management, and disaster response^3^.

The major techniques and approaches for LULC classification history start with manual classification and then with numerical and digital classification. Then it came that revolutionized artificial intelligence^4^. The first type of AI in LULC classification was Hard classification, which includes (traditional machine learning (unsupervised classification, Semi-supervised classification, supervised classification), and recent machine learning^5^. The second type of AI is soft classification (Fuzzy, Markov Random field theorem, and Object-oriented image analysis classification)^6^. Numerous challenges have arisen in LULC classification. The advent of artificial intelligence (AI) technologies has provided partial resolutions to some of these challenges. However, issues persist about the precision of LULC classification. Presently, hybrid methodologies have demonstrated efficacy in ameliorating the accuracy of LULC classification The introduction of a pioneering hybrid approach within this research exhibits promise in augmenting the accuracy of LULC classification^7^. The proposed approach in this study balances the learning rates for both components and optimizes the fusion strategy, which is essential. Training hybrid CNN models based on combined features from DenseNet121 offers a balance between computational efficiency and high-performance feature extraction^8^. The fusion of features spatial and spectral features extracted from DenseNet121 with the classifiers DT and XGBoost^9^. It presents an opportunity to create accurate models, making them suitable for various computer vision tasks. The efficacy of spatial and spectral feature extraction techniques depends on the characteristics of the remote sensing data, the complexity of the land cover classes, and the overall classification strategy^10^. The algorithms used in this research are effective choices for LULC classification since they can handle complex relationships in the data and make accurate predictions. In this regard, the fusion of spectral and spatial features derived through hybrid deep learning models has emerged as a revolutionary approach with great importance and relevance for LULC classification^11^. Hybrid deep learning-based spectral and spatial feature extraction is fundamental to LULC classification as it enhances the classification’s accuracy, effectiveness, and reliability. Integrating spatial and spectral features obtained from remote sensing images is highly capable of land change classification based on deep learning-based techniques^12^. Remote sensing image data often includes different spectral band data (blue, green, red, near-infrared) and spatial data (texture, shape, color) that contain valuable information for land cover and detection^13^. These hybrid models incorporate spatial, spectral, and temporal features from remote sensing data, allowing for a more comprehensive portrayal of the Earth’s development and enhancing classification precision Benefits of the Combined Approach, Enhanced Class Discriminative Power: The combined use of spatial and spectral features allows the classifier to capture intricate patterns and relationships in the data, increasing its ability to distinguish between land cover classes with identical spectral characteristics but distinct spatial structures. Robustness to Variability: Remote sensing data vary regarding atmospheric conditions, sensor specifications. The combined approach is robust to variability, as it leverages multiple sources of information^14^. The DT and XGBoost classifiers provide feature importance scores, allowing techniques to understand which features (spectral or spatial) contribute most to the classification decisions. This interpretability is valuable for analyzing and validating classification results.

The main contributions of this study are:

- Integration of complementary feature families: A hybrid spatial–spectral fusion framework combining handcrafted spectral descriptors with DenseNet121 deep spatial–spectral embeddings to improve discrimination of spectrally overlapping land-cover classes in arid environments.
- Six-band multispectral adaptation of DenseNet121: Modification of the first convolutional layer to accept six-channel Landsat 8 input (Blue, Green, Red, NIR, SWIR1, SWIR2), preserving full spectral information across visible and shortwave infrared bands.
- Enhancing Landsat 8 imagery of Najran city by utilizing the Linear Contrast Stretch (LCS) technique and Laplacian Filter.

The rest of the study is organized as follows: Sect.  2 extensively examines pertinent literature. The subsequent section, Sect.  3, expounds on the methodology, explains the process of acquiring data, pre-processing techniques, and the proposed hybrid deep learning architecture. Section  4 presents experimental findings, and Sect.  5 discusses and compares the system’s performance with previous studies. Section  6 presents conclusions.

## Related work

The section reviews a set of studies on LULC classification, discussing methodologies and results.

Recent research has illustrated various approaches to LULC classification, with the main differences in feature extraction methods and classification paradigms. Conventional machine learning methods are based on manually created spectral, statistical, and index-based features. For example, Aryal et al.^15^ systematically tested various feature schemes and classifiers and reported better performance with RF, achieving near-perfect accuracy; however, this performance is limited when using predefined features. Likewise, Ouma et al.^16^ compared tree-based and neural classifiers using multi-temporal data, demonstrating the superiority of RF but requiring post-classification fusion (FEI-FEO) to enhance performance, implying that single-model generalization is limited. Conversely, Dapke et al.^17^ emphasized the usefulness of SVMs with multiple kernels, but their performance is sensitive to kernel choice and does not model spatial context. Hybrid ML systems that incorporate feature engineering and state-of-the-art classifiers have demonstrated enhanced robustness. For example, Rajendiran et al.^18^ combined spectral indices and texture features with XGBoost and achieved high accuracy, albeit still relying on manual feature design. Research on complex terrains^19^ and hydrological use^8^ further supports the view that classifier performance is highly sensitive to feature quality and data characteristics. The framework in^20^ combines EfficientNet with optimization algorithms and achieves high performance, but at the cost of increased computational complexity and reduced interpretability. A thorough review^21^ suggests that 2D/3D CNNs are more effective than conventional methods because they can capture spatial-spectral correlations and do not require small datasets or considerable computation time.

Rui at el^22^. presented a feature-level fusion framework for LULC classification. It combined Landsat OLI images with varying cloud cover and a fully polarized ALOS-2 image in a study of the Karst Mountain region in Chongqing. Various spectral, texture, and spatial features were extracted, along with additional NDVI, elevation, and slope data. Methods include change index, Dematel for cause-effect analysis, Jaccard Similarity Index, and Adherence Index to study LULC transformation. Lucrêncio et al.^23^ aimed to map LULC classes in northern Mozambique from 2011 to 2020 using the Landsat time series and the Random Forest classifier in Google Earth Engine. Feature selection reduced redundant data. Shuai at el^24^. introduced Refine-EndNet, a deep feature fusion method tailored for hyperspectral and Synthetic Aperture Radar (SAR) data. It employed dynamic filter networks, attention mechanisms, and an encoder-decoder framework. Experimental results on ZY1-02D satellite hyperspectral and Sentinel-1 A dual-polarimetric SAR data demonstrate superior performance over conventional methods, making it promising for complex ground object classification in land cover ecosystems. Xilong et al.^25^ utilized long-term Landsat satellite images from Google Earth Engine (GEE) and the random forest classification algorithm to map land use changes in the NLR Basin from 1993 to 2022. Results indicate a declining wetland area and carbon storage due to land use changes, highlighting the importance of understanding wetland dynamics for regional carbon balance and ecology in the NLR Basin over 30 years. Sajjad et al.^26^ used remote sensing to analyze LULC changes and their impact on LST in Khanewal, Pakistan. Landsat images were processed with ERDAS Imagine and ArcGIS. Supervised classification revealed LULC changes in 1980, 2000, and 2020. Shengjie at el^27^. suggested the proposed method outperformed SVM and RF when tested on optical-SAR datasets and a hyperspectral dataset (University of Pavia). CNN leveraged spatial information for classification, significantly improving urban ground target accuracy. Gemechu et al.^28^ presented a study to forecast LULC change in the Vea catchment, Ghana, and its impact on irrigation water. They used the CA-Markov model for land-use predictions in 2038 and 2054 with Terrset software. The Relative Importance Index identified vital change drivers. The results indicate cropland growth from 181 m^2^ (2038) to 183 m^2^ (2054). Andrea at el^29^. aimed to develop and test an Object-Oriented classification approach that combines the Simple Non-Iterative Clustering (SNIC) algorithm for spatial cluster identification, the Gray-Level Co-occurrence Matrix (GLCM) for cluster textural indices, and two machine learning algorithms (Random Forest (RF) or Support Vector Machine (SVM) for final classification. PCA synthesizes textural information from seven GLCM indices into one band for classification. Ganesh at el^30^. A hybrid feature optimization algorithm combined with deep learning was introduced for improved LULC classification, which benefits wildlife habitat prediction and environmental quality assessment. It utilized Sat 4, Sat 6, and Eurosat datasets, pre-processed remote-sensing images, and employed a hybrid feature extraction approach. Seyd at el^31^. estimated Australian wildfire-affected areas using Sentinel-2 imagery and MODIS products in GEE. The results indicate that integrating local spatial information with PCM enhances classifier efficiency and improve ambiguity handling caused by landscape spectral confusion. Sergio et al.^32^ used Land cover images with labels for deep-learning model training. A fusion model integrates labels from multiple directions to assign a unique label to image sets. 2916 GLOBE images were labeled using an integrated model with minimal human input. Validation demonstrates a 90.97% label accuracy. The fusion model suits large land cover databases from RGB images. Anindita et al.^33^ aimed to track the periodic changes in LULC features from 1975 to 2018. To achieve this, the researchers employed the USGS-LULC classification method and a maximum-likelihood classifier algorithm utilizing satellite images.

Previous studies underscore a distinct emphasis on machine learning and deep learning-based classification research. While AI has shown promise in addressing LULC classification challenges, concerns persist regarding classification accuracy. The proposed model presented in this study is a hybrid approach that provides a novel idea not found in previous research. By integrating spatial and spectral features, this study aims to provide a robust solution for detecting and classifying various land changes, including urban expansion, land degradation, and vegetation dynamics, unlike other studies that rely primarily on only one spatial or spectral feature.

## Materials and methods

### Study area

**Fig. 1:**
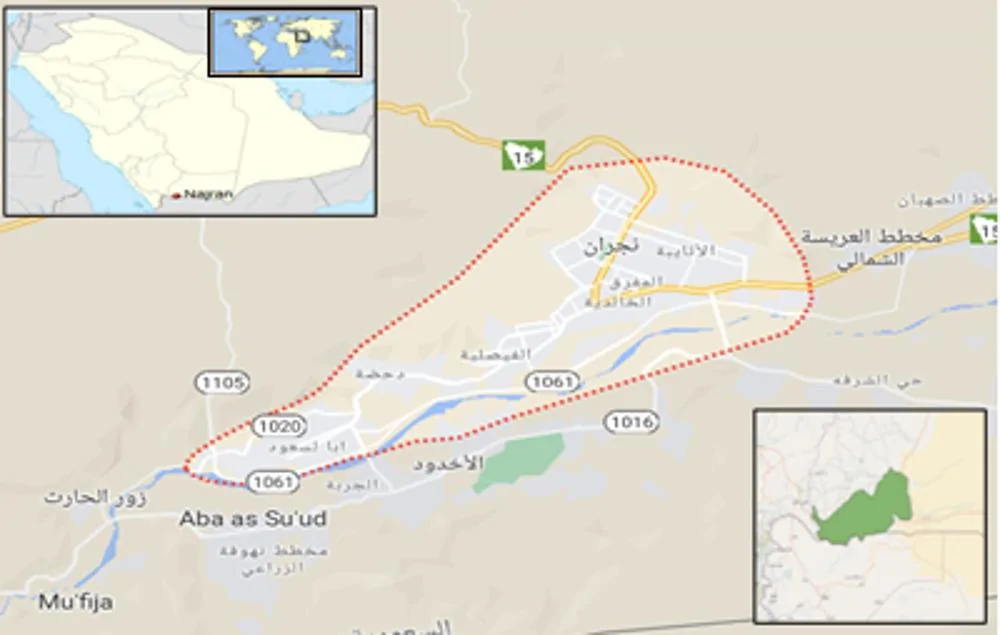
Geographic location of Najran city derived from Google Maps for spatial reference and study area orientation.

This study presents alterations in the capital of the Najran Region, situated in Najran City. The investigation utilizes Landsat 8 satellite imagery from 2020 to create maps. It functions as the administrative center of the Najran Province. Among the urban areas within the kingdom, Najran stands out with its remarkable population growth rate, earning it the status of a new town. The geographical coordinates of Najran are approximately 17.565604 latitude and 44.228944 longitude, denoted as 17° 33’ 56.1744” N and 44° 13’ 44” E^34^. Figure 1 presents a geographic overview of the study area derived from Google Maps, included to provide spatial context and facilitate reader understanding of the location of Najran city within the regional framework.

The region of interest in Najran is characterized by an arid and semi-arid environment, where land cover categories such as bare soil, sparse vegetation, and urbanized areas exhibit very similar spectral signatures. The ecological setup poses significant challenges for classifying LULC types due to the difficulty of differentiating spectrally homogeneous classes. In this regard, Najran constitutes an appropriate study site for assessing hybrid spatial-spectral feature extraction methods aimed at improving the classification discrimination in drylands.

### Source of data and data collection

Landsat 8 Operational Land Imager (OLI) and Thermal Infrared Sensor (TIRS) satellite images for the year 2020 were obtained from the United States Geological Survey (USGS) Earth Explorer database. The images were acquired during cloud-free conditions to ensure the quality of the data. The Level-1 data products were downloaded for the study area covering Najran. Each scene was in GeoTIFF format with a 30-meter spatial resolution.

The adoption of Landsat 8 sensor data is justified given its moderate spatial resolution and multispectral capabilities, which have proven highly effective in regional LULC classification studies, especially in diverse and resource-limited settings.

**Fig. 2:**
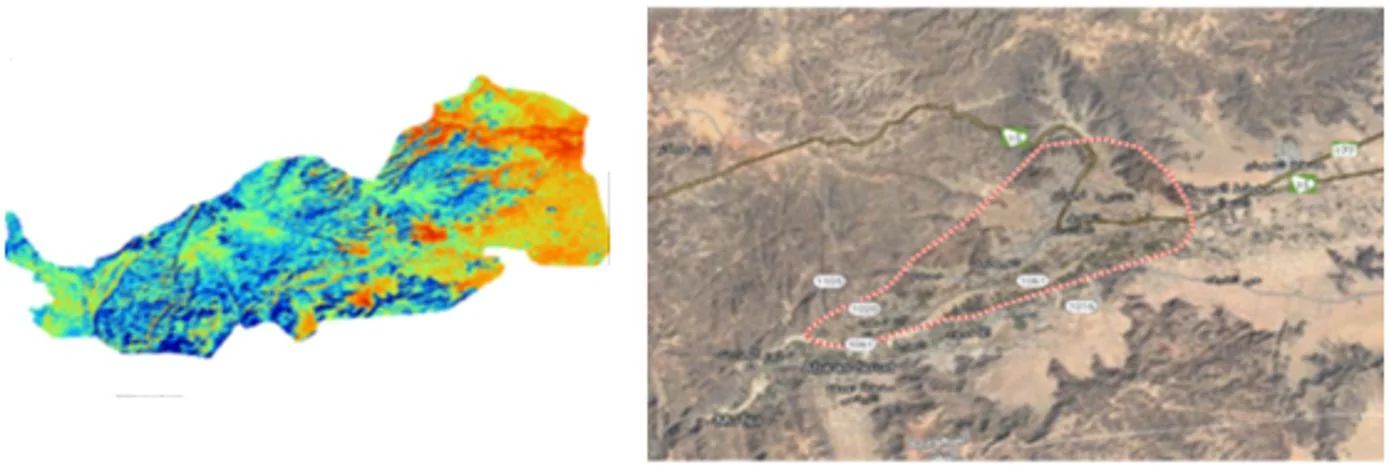
Georeferenced map of Najran City showing the study area boundary with coordinate grid, scale bar, and north orientation (WGS 84 projection).

The foundational map originates from survey images on SOI toposheets, scaled at 1:50000. The Landsat 8 sensor in 2020 facilitated the calibration and comparison of land features^35^. The data was procured through an operational land imager sensor on the Landsat 8 satellite. Data collection within the same region adheres to a recurring 16-day cycle. To make the map more interpretable in space and assure its accuracy, Fig. 2 shows georeferencing components, such as a coordinate grid, scale bar, and north arrow, based on the WGS 84 geographic coordinate system. The boundaries of the study area were defined using QGIS to specify their exact locations. Non-essential elements from Google Earth were deleted to make the map clearer and avoid unnecessary information. These amendments comply with remote sensing and GIS standards in cartography, which require geospatial referencing.

The satellite collects spatial reflectance information across eleven bands, encompassing a range of spectrum wavelengths. Seven bands capture data from the spectrum’s visible, near-infrared, and shortwave infrared portions. These chosen bands provide a spatial resolution of 30 m within a swath spanning 185 km. These bands were deliberately selected to form part of the land use dataset.

Atmospheric correction is a crucial step to remove the effects of the atmosphere from satellite images, enhancing the accuracy of subsequent analysis^36,37^.

Table 1 illustrates the stratified distribution of the dataset used for sustainable agriculture monitoring in Najran among the training (64%), validation (16%), and testing (20%) datasets. This type of partitioning is widely used in machine learning and remote sensing applications to ensure that model creation, tuning, and evaluation occur on mutually exclusive data.

In terms of sample counts, the total is 1,705,900; 1,091,776 for training, 272,944 for validation, and 341,180 for testing. Note that the distribution across the three datasets is proportional, which implies a systematic sampling process used by researchers. The proportional distribution of samples across the three datasets ensured that the classification problem was statistically representative, particularly given the presence of multiple LULC mapping classes.

**Table 1:** Dividing the dataset during the training, testing, and validation phases for monitoring agricultural sustainability in the city of Najran.

Class	Training 64%	Val 16%	Testing 20%	Total
Water Area	321,130	80,282	100,353	501,765
Agriculture	211,706	52,926	66,158	330,790
Built-up Area	219,821	54,955	68,694	343,470
Barren Land	295,302	73,826	92,282	461,410
Other Land	36,253	9063	11,329	56,645
Unclassified	7565	1891	2364	11,820
Total	1,091,776	272,944	341,180	1,705,900

### Pre-processing

The Surface Reflectance Code (LaSRC) is an algorithm offered by the United States Geological Survey that was used to rectify the satellite images. This algorithm removes atmospheric scattering and absorption, sensor calibration, and other radiometric effects to yield surface reflectance values that are more comparable across time and place^38^. Since the Landsat 8 images are very large, only a small portion of the images covering the Najran city area was selected. This subset minimizes computational load and focuses the analysis on the region of interest. Image mosaicing was then performed on the subset images to produce a continuous image of the study area. Radiometric normalization was performed to account for sensor sensitivity and changes in solar irradiance. This is done to ensure that pixel values across images can be directly compared and that spectral signatures are consistent throughout the dataset. Radiometric normalization reduces illumination and sensor response discrepancies that affect classification accuracy^39^. Image registration was performed to align all satellite images. This is done to ensure that images can be used in land-cover detection analysis. A robust registration system using ground control points (GCPs) and tie points was used to achieve accurate alignment^38^. To achieve uniformity across all images, they were resampled to a standard spatial resolution of 30 m. The nearest-neighbor algorithm was used for resampling to avoid unnecessary interpolation artifacts. Linear Contrast Stretch has greatly enhanced the imagery by applying image enhancement techniques. It uses LCS, which sharpens and smooths the image^30^.

**Fig. 3:**
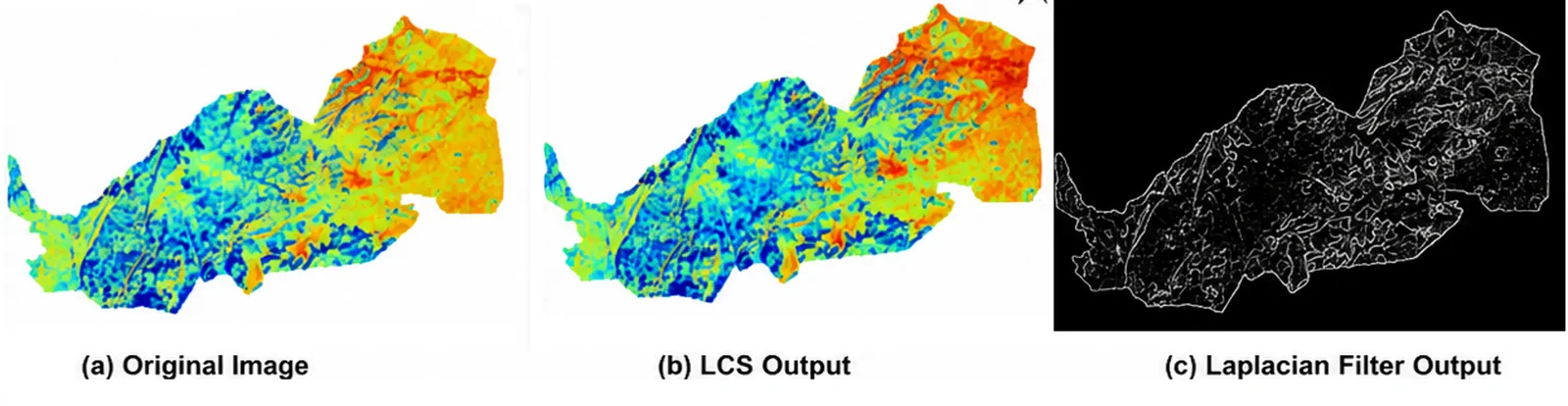
Comparative visualization of preprocessing stages applied to Landsat 8 imagery of Najran City: (**a**) original image, (**b**) LCS enhancement, (**c**) Laplacian edge detection for noise reduction and class boundary delineation.

LCS is a simple but effective method of enhancing satellite images. LCS is particularly useful in working with ideas whose possible data values are limited because of a small range between the lowest and highest possible values of the image, by using a linear contrast stretch to take into account the maximum and minimum brightness of the image without clipping. The LCS is of immense use when the data becomes saturated or clipped. Linear stretches are applied, resulting in minimal range expansion to span the entire range of values 0 to 255. This adjustment also enhances the darker and lighter parts, which boosts contrast in the image^40^. Figure 3 shows a map of Najran after enhancement by both LCS and the Laplacenial filter.

In turn, this improvement helps greatly to interpret the image visually. The addition of a Laplacian filter can significantly improve USGS (United States Geological Survey) map images. It is a critical filter used to highlight partition edges and make key features on the map clear. The Laplacian filter is useful for enhancing sudden intensity discontinuities, which in most instances may represent a boundary between different regions in the image. The Laplacian filter is important for representing partition edges in USGS map images^37^.

### Feature extraction

Deep feature extraction is an extremely good approach that helps the learned representations in deep neural networks from large datasets. Deep feature extraction facilitates knowledge sharing across existing models to new problems, reducing requirements for large labelled data and faster development of machine learning systems in applications. Deep feature extraction is a machine learning and computer vision technique for obtaining meaningful and abstract information from raw data, e.g., images or other high-dimensional data^41^. The process utilizes pre-trained deep neural models, typically CNN, to project input data into representative features. These features used in classification, object detection, image retrieval, etc^42^. Deep feature extraction, in concept, leverages the hierarchical representation learning ability of deep neural networks.

Two-dimensional CNNs have proven effective at learning spatial–spectral representations for multispectral remote sensing tasks, where multispectral bands are represented as input channel stacks. In this setup, convolution operations occur simultaneously across all spatial dimensions and the spectral-channel dimension. The proposed framework does not explicitly split spectral and spatial information into separate processing arms (e.g., DenseNet121)^43^. Instead, both types of information are acquired simultaneously in a hierarchical manner via convolution. The multispectral bands of Landsat 8 are presented as multichannel inputs composed of multiple convolution filters, each of which scans the spatial neighborhood and spectral channel dimensions. Early convolutional layers primarily detect local spectral responses and edge-level spatial textures, whereas successive dense blocks progressively capture higher-level spatial structures and nonlinear spectral relationships among the visible, near-infrared, and shortwave infrared bands^44^. This hierarchical representation learning allows the model to retain fine spectral variations while simultaneously modeling contextual spatial dependencies between neighboring pixels.

Consequently, the resulting feature maps contained spatial patterns, including texture, shapes, context, and the relationship in reflectance among different bands in the Landsat collection. The ability to learn from both spatial and spectral representations makes CNNs an effective choice for remote sensing problems, as they can efficiently model nonlinear class interactions.

For this study, DenseNet121 was chosen as the backbone CNN for extracting spatial-spectral features from Landsat remote sensing images of arid regions, as it better handles this type of image processing^45^. Unlike popular architectures such as ResNet or EfficientNet, DenseNet121 uses dense connectivity, meaning that each layer takes the outputs of all previous layers as input. Such an approach contributes to feature reuse and allows the retention of fine-scale spatial information, which is important for classification tasks in arid regions, where the spectral differences among classes (bare soil, urban development, and sparse vegetation) are not very pronounced. In addition, DenseNet121 addresses the vanishing-gradient problem and improves learning on moderately sized datasets. Thus, the model is better suited for remote sensing with a relatively small number of examples^46^.

Spectral and spatial characteristics, harnessed through the advanced hybrid deep learning architectures, assume a central role in LULC due to their profound capacity to elevate the classification process’s precision, efficiency, and resilience. Integrating spatial and spectral attributes from remote sensing data yields remarkable efficacy in classifying land transitions using deep learning models. Remote sensing data encompasses diverse spectral bands (red, green, blue, near-infrared, etc.) and spatial attributes (texture, form, and context). These collectively furnish invaluable insights for analyzing land cover and land use alterations. These hybrid models seamlessly integrate spatial, spectral, and temporal attributes extracted from remote sensing data, enabling a comprehensive portrayal of the earth’s transformations and yielding advancements in classification accuracy^47^.

The proposed approach does not involve extracting spatial and spectral information using two separate branches. Instead, both types of information are learned in parallel by DenseNet121, with six-band multispectral images as input. On the other hand, handcrafted spectral indices are generated separately as auxiliary features.

#### Spectral features

Spectral features obtained from DenseNet121 offer a robust combination to most image analysis and classification tasks. DenseNet121 is CNN architecture defined by depth and dense connectivity, hence best suited to capture subtle patterns and features in images^12^. When applied to spectral data multi-spectral, DenseNet121 obtains useful spectral information that classifiers use for various applications, from remote sensing. DenseNet121 is a variant of the DenseNet model characterized by its dense block structure^48^.

Each layer receives input from all the preceding layers in a dense block, which encourages feature reuse and gradient propagation throughout the network. The dense connectivity mechanism is particularly beneficial for preserving spectral features because feature maps generated in earlier layers are directly concatenated with those in subsequent layers rather than discarded. This architecture strengthens gradient propagation and allows low-level spectral responses to remain accessible throughout the network depth. Consequently, DenseNet121 can better discriminate spectrally similar classes commonly observed in arid and semi-arid environments, where subtle reflectance differences between barren land, urban surfaces, and sparse vegetation are difficult to distinguish using conventional convolutional architectures. This new structure enables DenseNet121 to learn low-level and high-level features in an image, thus being highly efficient in feature extraction^27^.

When applied to spectral data, DenseNet121 handles a spectral band or channel like RGB channels when dealing with normal image data. In the proposed architecture, the multispectral Landsat 8 bands were arranged into a multi-channel tensor and fed into the DenseNet121 model as input. Although DenseNet121 uses two-dimensional convolution filters, during convolution, the filters scan across the entire channel dimension, thereby simultaneously extracting spatial-spectral features. Therefore, the convolution kernels can simultaneously extract spatial information within a region and spectral interactions between different bands, including visible, near-infrared, and short-wave infrared.

DenseNet121 learns hierarchical abstractions of the spectra by repeating convolution and dense feature propagation. The shallow layers are sensitive to low-level spectral reflectance differences between Landsat bands, whereas deeper layers progressively learn complex nonlinear spectral signatures related to vegetation vigor, soil composition, urban materials, and moisture conditions. Each dense layer has access to the outputs of all previous layers, so low-level spectral responses can be easily retrieved throughout the network’s depth, reducing information loss and enhancing discrimination between spectrally overlying classes frequently seen in arid and semi-arid environments^49^.

The DenseNet121-only learned deep features without handcrafted indices, whereas hybrid models combined both, as in conventional machine learning techniques. Low-level spatial features, such as edges and local textures, were mostly learned by the earlier convolutional layers, whereas higher layers learned progressively higher-level contextual and semantic information, such as the spatial organization of barren surfaces, vegetation distribution patterns, and urban morphology. This hierarchical learning process enhances discrimination between overlapping classes in a spectrally similar manner, as is typical in arid environments.

While the convolution layers at shallower depths mainly extract low-level spectral features, including reflectance and edge features, deep dense blocks gradually build high-level spatial-spectral features by leveraging hierarchical feature propagation and dense connectivity. It allows the network to learn about spectral patterns and inter-relations in the data to extract spectral features specific to the task^50^.

Handcrafted spectral indices should not be confused with deep spectral representations obtained using DenseNet121. The indices, such as NDVI, EVI, NDWI, NDMI, and Tasseled Cap, were not generated internally by the DenseNet121 architecture. They were calculated externally in advance using mathematical formulas based on remote sensing theory and then used as an additional set of spectral descriptors. In contrast, deep spectral-spatial representations were automatically learned from multi-spectral bands using convolutional filters applied to each channel separately. This means that the features extracted from CNNs are data-driven hierarchical abstractions and not handcrafted indices. Deep spectral-spatial representations were derived independently by the Deep Net, while conventional spectral indices were derived prior to learning and used as an additional descriptor^51^.

Spectral Signatures: DenseNet121 learns to recognize certain spectral signatures corresponding to different materials or classes of hyperspectral data. Spectral signatures are the typical patterns of reflectance or emission at unique wavelengths.

Spectral Texture: The network captures textural information in the spectral data. It is essential in remote sensing applications where the texture of the land surface provides valuable information about land cover or land use.

Spectral Bands of Interest: DenseNet121 emphasizes specific spectral bands that are particularly relevant for the classification task. It is valid for dimensionality reduction and improved computational efficiency.

Spatial-Spectral Features: If spectral data is associated with spatial information (multi-spectral images), the network extracts spatial-spectral features, considering the spectral content and spatial relationships between neighboring pixels.

The proposed framework does not restrict feature representation to handcrafted spectral indices for classification only. In contrast, a spatial-spectral feature vector was combined instead of the deep spatial-spectral representations automatically extracted from the spectral patches of multispectral Landsat images using DenseNet121 and conventional handcrafted spectral descriptors, such as NDVI, NDWI, EVI, NDMI, Tasseled Cap components, raw bands, and NIR reflectance values. The DenseNet121 design learned hierarchical representations of the data in both the spatial and spectral dimensions by performing convolutions in parallel across both dimensions. This resulted in deep features extracted from the images that represented spatial texture, contextual information, edge structures, object morphology, and nonlinear spectral interactions between the visible, NIR, and SWIR bands. These deep representations were then compressed and appended to hand-designed spectral indices to produce a final feature space for the classifiers (DT and XGBoost).

After removing spectral features with DenseNet121, classifiers are employed to categorize or label the data based on these features. The choice of classifier varies depending on the problem and dataset^52^. Combining spectral feature extraction using DenseNet121 and subsequent classification techniques enables the practical analysis of spectral data for various applications. This approach leverages deep learning and spectral data analysis strengths to provide accurate and valuable insights from complex spectral datasets^53^.

Spectral feature extraction is pivotal in LULC classification when utilizing remote sensing data and satellite imagery. Spectral features enable the capture of information about the distinct reflectance characteristics exhibited by various land cover categories. This information is subsequently employed to discriminate and categorize this land cover types^36^.

This work begins by obtaining satellite imagery or remote sensing data pertinent to the area of interest. Pre-process the acquired data to correct for atmospheric interference, radiometric calibration, and geometric alignment. It identifies and designates representative regions within the imagery corresponding to different land cover classes. These selected ROIs will be the basis for extracting training samples for each category. It is imperative to choose diverse areas that adequately encapsulate the spectral variability characteristic of each land cover type^54^. It extracts sufficient pixels from the previously designated ROIs for each land cover category.

These extracted pixels will be utilized as training samples for the classification algorithm. It is crucial that these samples accurately represent the spectral variability inherent within each respective class^55^. Extract spectral features from each pixel within the training samples. Commonly employed spectral bands, indices, and transformations used as features encompassed in this work as follows^56^: The aforementioned spectral indices were derived prior to obtaining deep spectral-spatial features and therefore had physical significance for vegetation vigor, soil moisture, soil water content, and surface reflectance. By combining them with deep representations, an enhanced representation of the data can be developed that includes both human-driven spectral knowledge and automatic learning.

- Raw spectral bands (Blue, Green, Red, Near-Infrared, SWIR1, SWIR2).
- Vegetation indices (NDVI, EVI).
- Soil indices (NDMI, NDWI).
- Tasseled Cap transformation components.

As one can see, both handcrafted and deep features are complementary, since the former include physically interpretable information, while the latter involve learning complex hierarchical representations. They are combined prior to classification.

#### Spatial features

Spatial features extracted from DenseNet121 with the classifiers offer a powerful approach for various computer vision tasks, including image classification, object detection, and semantic segmentation. DenseNet121 is a CNN architecture that excels in feature extraction due to its densely connected blocks, encouraging feature reuse and propagation throughout the network^8^. When combined with classifiers, DenseNet121 extracts spatial features that are highly informative and discriminative^57^. DenseNet121, designed based on densely connected blocks and classifiers, extracts spatial features encompassing extensive information from input images. The features are the backbone of understanding and interpreting spatial relationships and structures of the visual data, and therefore DenseNet121 is a universally available choice for various computer vision tasks^58^.

Spatial features are extracted using DenseNet121 with classifiers^41^.

DenseNet121 begins with convolutional layers to extract low-level features from the input image. These layers are responsible for extracting elementary patterns and edges^25^.

One of the key features of DenseNet121 is that it consists of densely connected blocks made up of multiple convolutional layers stacked on top of one another. Every layer receives input from all the previous layers in these blocks, creating a dense connectivity pattern. It enables the network to be capable of extracting low-level and high-level features efficiently.

At each highly connected block, feature maps are generated. The spatial features in the proposed framework comprise the structure and context extracted from the neighboring pixels within the Landsat 8 image. In the remote sensing context, spatial features encompass edges, texture statistics, boundaries, geometry, spatial relations, continuity, and neighborhood structure. In remote sensing classification tasks, spatial information is critical because it can distinguish between land-cover classes with similar spectral signatures but different spatial patterns, such as built-up land and barren land in drylands. In DenseNet121, spatial information in the form of edges, corners, and textures is detected by shallow convolutions, whereas deeper dense layers detect more semantic high-level representations related to urban buildings, agricultural field layout, road systems, and diverse landscape morphology. The dense network structure enables hierarchical reuse of spatial representations within layers, allowing both the preservation of detailed contextual information and the learning of broader spatial dependencies.

In DenseNet121, spatial feature extraction is performed using a hierarchical convolutional receptive field that increases in depth. The early layers focus on local spatial features, including edges, corners, and texture variations, whereas deeper dense blocks are used to identify more complex spatial patterns, such as urban morphology, agricultural field layouts, road networks, and continuity patterns in land cover. This spatial abstraction, which increases gradually, allows the model to discriminate between land-cover classes with similar spectral appearances but different spatial patterns and contexts^44^.

**Fig. 4:**
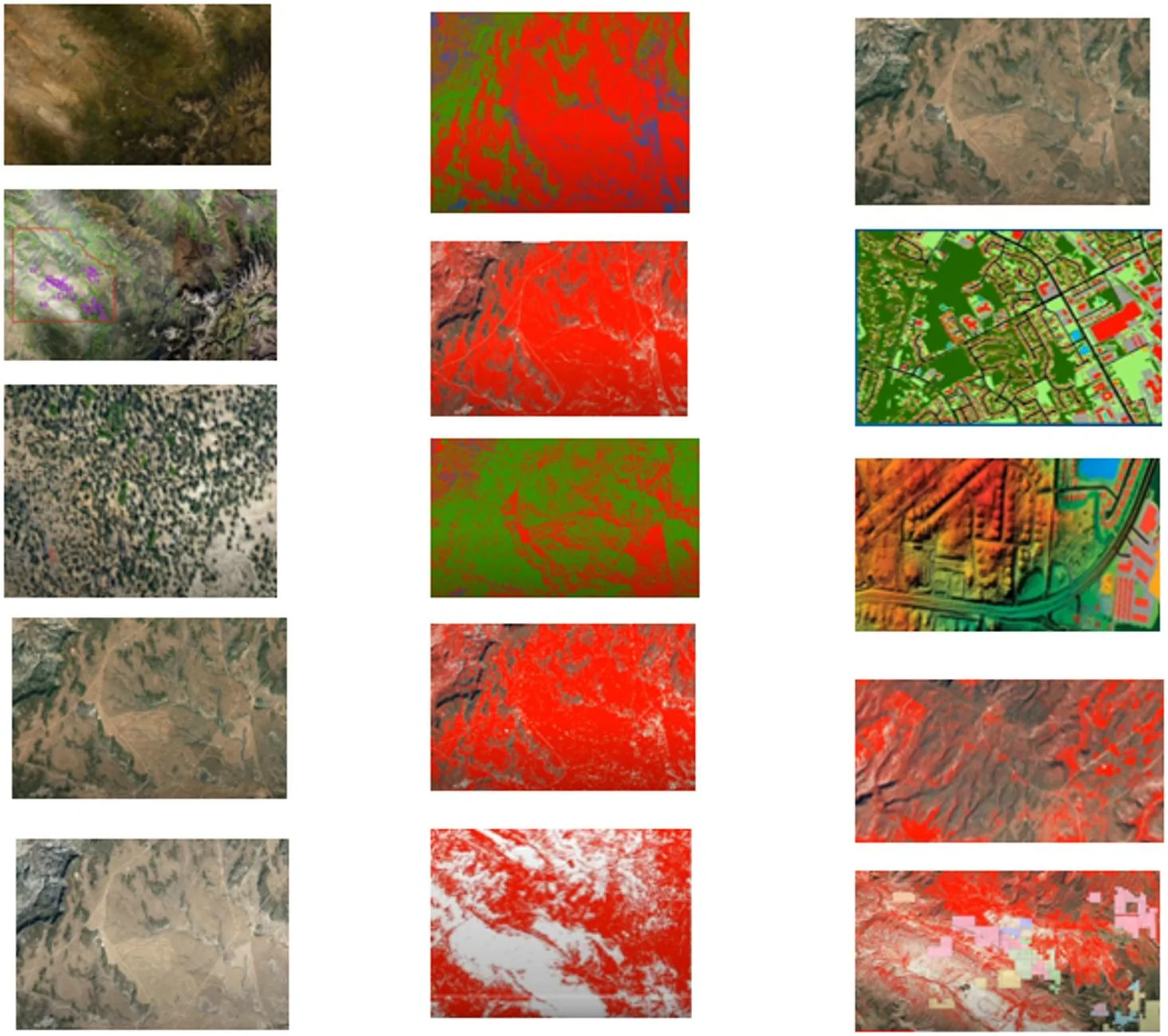
Hierarchy of spatial feature representations derived from Landsat 8 satellite imagery using DenseNet121. Low-level spatial features, such as edges and textures, are captured in early convolutional networks, whereas higher-level spatial features, such as the morphological structure of urban areas, agricultural field layout, road layout, and relationships between land cover classes, are learned in denser blocks.

The feature maps describe various aspects of the input image, from simple textures to complex parts of objects. Feature maps of previous layers receive local details^7^, while later layers capture more abstract and global information. Figure 4 shows the hierarchical spatial features at various levels of DenseNet121.

### Machine learning classifiers

The researchers employed a set of machine learning classifiers, DT and XGBoost, to transform the derived features into understandable classification results. All the classifiers are computationally efficient and support imbalanced data. By comparing classifier performance, the research aimed to choose the top-performing model to classify the LULC classes in Najran city efficiently.

Prior to classification, deep spatial–spectral representations were extracted using a modified DenseNet121 architecture adapted for six-channel multispectral Landsat 8 imagery (Blue, Green, Red, NIR, SWIR1, SWIR2).

Concatenated feature vectors consist of 13 handcrafted and 1024 deep features (total number of dimensions equals 1037).

#### DT classifier

DT Classifier is one of the most popular machine learning techniques applied for LULC classification in geospatial and remote sensing applications. A supervised learning method labels land cover categories with class labels from input attributes: satellite image spectral bands, terrain parameters, and any available information. The DT classifier was used for its ability to partition the feature space into interpretable decision regions, which is useful for understanding classification behavior in LULC mapping. Each node in the tree represents a decision point, and each branch represents a possible outcome^5^. The leaves of the tree correspond to the final class labels.

The DT is trained on a dataset with spectral indices, Normalized Difference Vegetation Index (NDVI), and Normalized Difference Water Index (NDWI), as well as Near-Infrared (NIR) reflectance values.

Deep spatial–spectral features extracted by the modified DenseNet121 architecture were also provided to the DT classifier, along with the handcrafted spectral descriptors. The adapted DenseNet121 model processed six-channel Landsat multispectral imagery (Blue, Green, Red, NIR, SWIR1, and SWIR2), enabling the network to encode spatial texture, neighborhood context, geometric structures, and inter-band spectral relationships. Combined handcrafted spectral indices with automatically learned deep spa-tial–spectral representations were used to form the final DT input vector. This mixed representation improved the separation of spectrally similar classes, such as barren land, sparse vegetation, and urban surfaces in the arid landscape of Najran.

The algorithm builds the tree to minimize impurity or maximize information gain at each split. The DT offers an interpretable model easily visualized and understood by domain experts. DT learns complex, non-linear relationships in the data, which are well-suited for various LULC classification tasks. They perform well with big data and are computationally efficient for training and inference.

DT also evaluates the relative importance of various features in distinguishing land cover types. But DT is prone to overfitting, particularly when it becomes deep. RF, or Gradient Boosting, that blends many DTs to enhance accuracy and generalization. Overall, a DT Classifier is an efficient tool for LULC classification that facilitates automation of land cover mapping using input data and a collection of decision rules. It employs a seemingly intuitive method of land cover type categorization, which is beneficial for applications such as remote sensing and environmental monitoring^59^.

Stratified 5-fold cross-validation was applied only for hyperparameter optimization and model selection to guarantee that the training and validation sets had non-overlapping spatial regions (geographically disjoint patches) using fixed-size spatial patches (tiles). To ensure comparability, all models were tested using the same data and cross-validation splits. The independent test set (20% of the total set) was not used for cross-validation or training and was used only for the final performance evaluation. In this section, all experiments are based on separate test sets. The number of test samples per class is presented in Table 6. To assess stability, three runs with different random seeds were performed, and the results are presented as mean ± SD.

#### XGBoost classifier

XGBoost (Extreme Gradient Boosting) is a popular machine-learning classification and regression algorithm. Equations 2–7 for the XGBoost classifier are described as follows: For a dataset with N samples and M features, Initializing the model with a constant value, typically the mean of the target values for regression problems or the log(odds) of the class distribution for classification problems^60^.

The XGBoost algorithm is based on the gradient-boosting framework of decision trees. It enhances the performance of classification tasks by minimizing a regularized loss function. The application of the XGBoost algorithm to the LULC classification task was based on its ability to capture nonlinear interactions.

This equation outlines the core steps of the XGBoost classifier algorithm, which combines the predictions of multiple DTs to make accurate predictions for classification tasks^61^.

The hybrid spatial-spectral feature space applied to the DT model was also used to train the XGBoost classifier. In particular, these input variables comprised deep spatial-spectral representations derived from DenseNet121, handcrafted spectral indices, and multispectral reflectance variables. Such a fused representation enabled XGBoost to capture complex nonlinear relationships among spatial context, texture, and spectral signatures, thereby enhancing the robustness of classification in heterogeneous arid and semi-arid landscapes.

#### Hybrid approach for improved classification

This strategy aimed to harness the complementary information in spatial and spectral domains to enhance classification performance. The spatial features provided insights into the arrangement and relationships of objects on the ground, while the spectral features characterized the unique spectral signatures of different land cover types.

The hybrid approach integrates the spatial feature extraction strengths of DenseNet121 with DT and XGBoost’s classification strength. Employ the pre-trained Dense-Net121 model to extract spatial features from remote sensing images. The features capture spatial patterns, textures, and contextual information, significantly enhancing classification accuracy. Meanwhile, it preserves spectral components from the original data, keeping the important spectral signatures of different land cover types and materials. The spectral and spatial characteristics are combined into a hybrid feature space, creating an enriched representation of the data that leverages the spatial and spectral information^30^.

Specifically, the hybrid feature fusion process consisted of concatenating deep spatial-spectral embeddings derived from DenseNet-121 with handcrafted spectral variables, including NDVI, NDWI, EVI, NDMI, Tasseled Cap components, and raw multispectral reflectance bands. This integration approach enabled the classifiers to leverage both the high-level contextual representations automatically learned from the imagery and the physically interpretable spectral indices. Spatial–spectral fusion has been widely reported in the remote sensing literature to improve the discrimination between similar land-cover categories and to enhance the stability of land-cover classification in complex dryland environments.

**Fig. 5:**
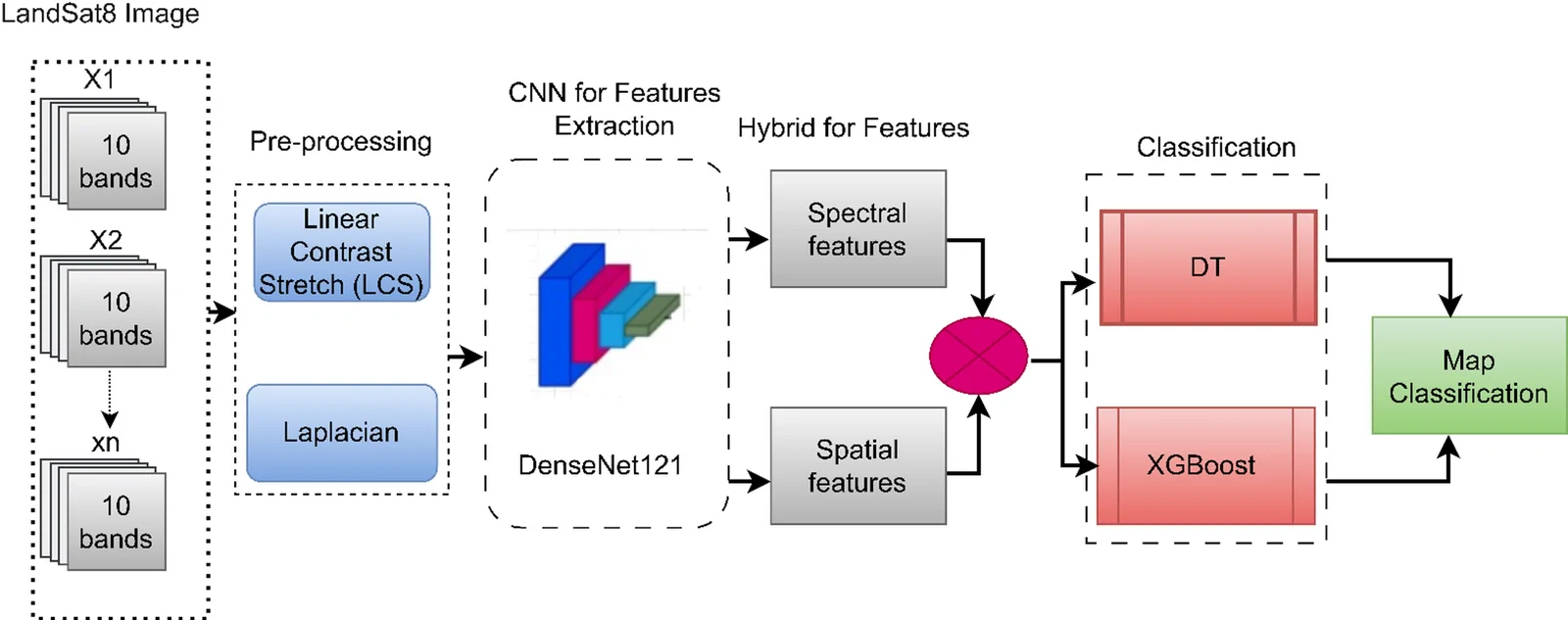
Proposed hybrid spatial-spectral classification framework for Najran LULC mapping.

DT provides transparency and insight into the classification process, while XGBoost offers improved predictive performance and robustness. Combining spatial and spectral features with DT and XGBoost, our hybrid approach aims to achieve higher classification accuracy and improved generalization on remote sensing datasets. This fusion of techniques capitalizes on the complementary nature of spatial and spectral information, allowing us to extract the most valuable insights from multi-modal remote sensing data. Figure 5 shows the proposed hybrid approach^62^.

DT and XGBoost were selected as methods owing to their advantages based on the characteristics of the extracted feature space and the nature of the LULC classification task. First, the obtained hybrid feature space using DenseNet121 was highly dimensional and could not be linearly separated. Feature space tree-based algorithms should be used to solve this problem. The main advantage of DT is that it can provide an interpretation of the model’s decisions. This is important for applications in agriculture and environmental studies, where the interpretability of the algorithm is critical. One of the disadvantages of DT is that it tends to overfit the data, especially when the dataset is imbalanced. XGBoost was selected to solve this problem because, as an ensemble learning method, it creates many different regularized trees that generalize better and help find nonlinear interactions between features. Moreover, compared with Random Forest, XGBoost provides more control over the regularization and boosting processes.

The categories have been seen in the variations in land cover in Najran City. The QGIS software also produces the confusion matrix, which displays the percentage of pixels changing from one kind to the next, which are Water Area, Built-up Area, Agriculture, Barren Land, and Other Land, the six samples for the six parameters that make up the input layers for model processing. These parameters are explained in Table 2. The samples are based on the RGB color composites of Landsat8 images in the Vegetation class (red pixels in the RGB color composite RGB = 432).

**Table 2:** Description of the LULC classes and their spectral characteristics derived from Landsat 8 imagery for Najran City.

LCLU Class	Description
Land Area	The area in square kilometers of the land-based portions of conventional geographic regions is called the land area, which is the population of peo-ple. It does not contain buildings, streets, parks, roads, or buildings that have crashed like this.
Built-up Area	Built-up areas: Large buildings, small buildings, settlements, transporta-tion, land, or places containing people like banks, schools, hospitals, etc.
Agriculture	Space containing crops, fields, sparse grassland, a Temperate steppe, and a Temperate meadow.
Bare Land	Bare soil, bare rocks, and land do not contain people like the desert.
Water area	This area is a lake, sea, river, etc.
Other Land	Unknown Land

**Fig. 6:**
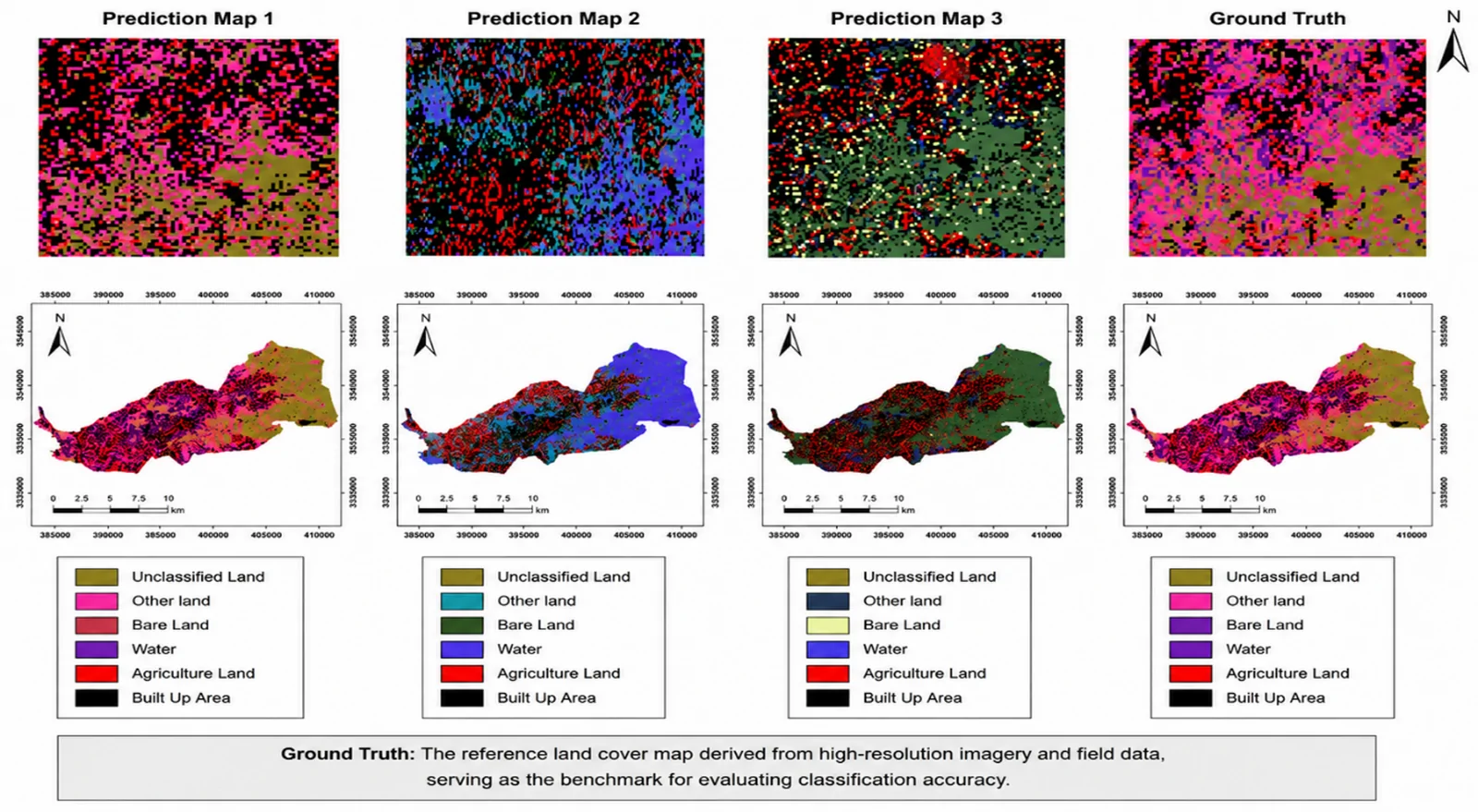
Comparative visualization of LULC classification results for Najran City: (a) Ground Truth (GT) map derived from the independent testing dataset (20%), (b) DenseNet121 classification, (c) DenseNet121 + DT, and (d) DenseNet121 + XGBoost. The inclusion of the GT map enables direct visual assessment of classification accuracy and spatial consistency.

Figure 6 compares and assesses LULC classification using DenseNet121, combining spatial and spectral features extracted by DenseNet121 with DT, and combining spatial and spectral features extracted by DenseNet121 with XGBoost, considering the training optimization model’s parameterization technique and the case study area’s geographic distribution of land cover. The landscape is flat in the southern section. It is primarily agricultural terrain and is the center of Najran City’s agricultural production. Building land and aquatic bodies are heavily populated in the eastern urban/suburban areas, and arable land and building land are scattered throughout. Moreover, a sizeable body of water dominates the heart of Najran city. For an unbiased and rigorous evaluation process, the ground truth (GT) dataset was obtained exclusively from the independent test set, which comprises 20% of the entire dataset.

Thus, the hybrid method should be understood as the proposed approach to fusing spatial-spectral features, rather than as a new architectural design for a deep model. Specifically, such a method implies fusion of complementary feature families, namely, handcrafted indices and deep spatial-spectral features (DenseNet121).

#### Model validation, reproducibility, and implementation settings

To make our method transparent and reproducible, we employed a stratified hold-out validation split. The total number of samples in the dataset was 1,705,900, and each sample represented 4,500 m² of the ground surface area. Stratification was performed at the class level, such that all subsets maintained the same relative distribution of all LULC classes.

The testing data were not included in any steps prior to the final calculation of metrics and remained entirely independent throughout the process. The model was chosen based solely on validation accuracy, while metrics such as accuracy, precision, recall, F1 score, and specificity were calculated on the independent test data. To improve stability, three independent tests with different random seeds were run and averaged.

Stratified 5-fold cross-validation was applied only for hyperparameter optimization and model selection to guarantee that the training and validation sets had non-overlapping spatial regions (geographically disjoint patches) using fixed-size spatial patches (tiles). To ensure comparability, all models were tested using the same data and cross-validation splits. The independent test set (20% of the total set) was not used for cross-validation or training and was used only for the final performance evaluation. To assess stability, three runs with different random seeds were performed, and the results are presented as mean ± SD.

DenseNet121 was selected as the deep feature extractor because of the network’s densely connected architecture, which leverages features and enables better gradient propagation, thereby making the extraction of hierarchical spatial–spectral features from image patches more effective. DenseNets are a class of network architectures proposed to enhance information exchange between layers and improve training efficiency.

DenseNet121 was chosen for its ability to represent multispectral remote sensing data. Dense connections enable efficient feature reuse and gradient flow during fine-tuning while retaining low-level spectral information. This approach is well-suited to processing Landsat 8 multispectral images, which are characterized by highly correlated spectral bands.

In this study, DenseNet121 serves as a feature extractor rather than a classifier; that is, its primary task is to learn hierarchical spatial-spectral representations within a hybrid feature-fusion pipeline. The extracted features were then combined with handcrafted spectral indices and classified using decision trees and XGBoost.

Multispectral bands derived from the Landsat 8 OLI sensors were selected because they offered 30 m resolution. This was deemed sufficient for LULC classification using remotely sensed imagery. For implementation, the image patches were resized to 224 × 224 pixels to match the input dimensions of DenseNet121. The data augmentation process was limited to the training images and included random horizontal and vertical flips, rotations, zooms, and brightness changes.

Decision trees were used for their interpretability and ability to derive decision rules for LULC classification. However, to avoid overfitting, the tree depth, minimum split size, and minimum leaf size were all limited. XGBoost was selected as the ensemble classifier owing to its ability to perform regularized gradient-boosted decision trees and its wide adoption in structured data classification, owing to its computational efficiency, scalability, and inherent regularization techniques. This hybrid framework combines hierarchical DenseNet121 feature extraction with gradient-boosted tree classification.

The implementation of these models used Python with TensorFlow and Keras for DenseNet121, while the machine learning classification model used Scikit-learn and XGBoost libraries. Both libraries provide tools to train, test, and deploy deep learning and ensemble methods, such as gradient boosting. This implementation allows other researchers to reproduce the experiment and conduct comparative research on other datasets from arid and semiarid agricultural regions.

The DenseNet121 architecture’s hyperparameters were optimized to strike a suitable balance among convergence speed, strong generalization, and reasonable computational cost during training, as shown in Table 3. The input patch size was set to 224 × 224 pixels with 6 spectral bands (Blue, Green, Red, NIR, SWIR1, SWIR2). The first convolutional layer of DenseNet121 was adapted to accept 6-channel input instead of the original 3-channel RGB input. Weights were initialized using ImageNet pretrained parameters for the corresponding input channels. The full model was subsequently fine-tuned on the Landsat dataset. We selected the Adam optimizer because it adapts its learning rate and effectively handles sparse gradients. The initial learning rate was set to 1 × 10⁻⁴, with a ReduceLROnPlateau scheduler to enable stable convergence by decreasing the learning rate when validation performance began to plateau. A batch size of 32 was selected as a compromise between memory constraints and gradient stability. Finally, training was performed for up to 50 epochs to allow sufficient learning. To prevent overfitting, early stopping was used with respect to the validation loss, with a patience of 10 epochs, restoring the model weights that produced the best performance (the lowest validation loss). Categorical cross-entropy was used as the loss function because it is appropriate for multi-class LULC classification. In addition, three independent runs of the experiment were conducted to strengthen the robustness and reproducibility of the reported results by reducing the effect of randomization.

The original DenseNet121 was trained using ImageNet (input: 3-channel RGB). The input imagery was a six-channel Landsat 8 OLI multispectral image consisting of Blue, Green, Red, NIR, short-wave infrared 1 (SWIR1), and short-wave infrared 2 (SWIR2).

Input tensor specification: The input tensor size was (batch_size, 224, 224, 6) with channel order [Blue, Green, Red, NIR, SWIR1, SWIR2].

Normalization: Normalization was performed independently for each band by computing statistics from the training data, ensuring each band had a mean of zero and a standard deviation of one.

First-layer changes: The first layer of DenseNet121 was modified to accept a 6-channel input rather than a 3-channel RGB input. The weights corresponding to RGB channels were initialized using pretrained ImageNet parameters.

**Table 3:** Training configuration and hyperparameter settings for the DenseNet121 model.

Reviewer-required item	Adopted setting
Input patch size	224 × 224 pixels
Input channels	6 multispectral bands (Blue, Green, Red, NIR, SWIR1, SWIR2); first convolutional layer adapted to accept 6-channel input; pretrained ImageNet weights partially transferred to RGB-compatible channels
Data augmentation	Random horizontal flip, vertical flip, ± 20° rotation, zoom range 0.10, brightness range 0.80–1.20
Dataset split	Stratified 64% training, 16% validation, 20% independent testing
Optimizer	Adam
Initial learning rate	1 × 10⁻⁴
Learning-rate schedule	ReduceLROnPlateau; factor = 0.5, patience = 5 epochs, minimum LR = 1 × 10⁻⁶
Batch size	32
Maximum epochs	50
Early stopping	Validation loss monitored; patience = 10 epochs; best weights restored
Loss function	Categorical cross-entropy
Model-selection criterion	Lowest validation loss and highest macro-F1 score
Final evaluation	Independent test set only
Independent runs	3 runs with different random seeds
Hardware	Lenovo laptop, 11th-generation Intel Core i7, 16 GB RAM, 8 GB GPU
Approximate training time	DenseNet121 feature extraction/fine-tuning: ~5–7 h

The hyperparameters of the DT and XGBoost classifiers were tuned to achieve optimal classification accuracy and avoid overfitting, as shown in Table 4. For the DT model, constraining the maximum tree depth to 20, the minimum samples to split to 10, and the minimum samples per leaf to 5 reduced overfitting owing to increased variance. Using Gini as the impurity measure for tree nodes enabled us to identify the most effective splits and create efficient decision boundaries.

For the XGBoost classifier, various regularization parameters were used to improve performance and enhance generalization. A maximal tree depth of 8 and a learning rate of 0.05 provided sufficient flexibility to capture complex relations without overfitting. Using 300 estimators and random subsamples of features and observations during the training procedure (0.80 and 0.80, respectively) improved model generalization and reduced overfitting risk. The use of regularization parameters, such as gamma (0.10), L1 penalty (0.10), and L2 penalty (1.00), decreased overfitting and increased robustness when working with highly dimensional feature spaces generated using the DenseNet121 model. The multi-softprob objective function allows the generation of probabilistic predictions for multi-class classification tasks.

**Table 4:** Hyperparameter configuration for Decision Tree (DT) and XGBoost classifiers.

Parameter	Decision Tree	XGBoost
Criterion/objective	Gini impurity	multi: softprob
Maximum depth	20	8
Number of estimators	Not applicable	300
Learning rate	Not applicable	0.05
Minimum samples split	10	Not applicable
Minimum samples per leaf	5	Not applicable
Subsample	Not applicable	0.8
Column sample by tree	Not applicable	0.8
Gamma	Not applicable	0.1
L1 regularization	Not applicable	0.1
L2 regularization	Not applicable	1
Class imbalance handling	Class-weighted training	Class-weighted/sample-weighted training
Random state	Fixed seed	Fixed seed
Approximate training time	~ 20–35 min	~ 1.5–2.5 h

#### Feature specification, fusion strategy, and reproducible experimental configurations

To address the issue of feature isolation and reproducibility, this section explicitly specifies: (1) which features are handcrafted vs. learned, (2) their dimensionalities, (3) the concatenation mechanism, and (4) controlled ablation configurations.

Feature families: Table 5 categorizes all features used in the proposed pipeline.

**Table 5:** Feature families: handcrafted spectral indices vs. deep learned features.

Feature Family	Type	Extraction Method	Dimensionality
Raw spectral bands (Blue, Green, Red, NIR, SWIR1, SWIR2)	Handcrafted	Pixel extraction from Landsat 8	6
Vegetation indices (NDVI, EVI)	Handcrafted	Mathematical formula	2
Soil/Water indices (NDWI, NDMI)	Handcrafted	Mathematical formula	2
Tasseled Cap components	Handcrafted	Linear transformation	3
Deep spatial–spectral features	Learned	DenseNet121 (6-channel input)	1024

Total handcrafted dimensionality: 6 + 2 + 2 + 3 = 13 features.

Total learned dimensionality: 1024 features (extracted from the final global average pooling layer before classification).

Total fused feature vector dimensionality: 13 + 1024 = 1037 features.

Concatenation mechanism: The fusion process follows Eq. 31:

$${\mathrm{F}}_{{{\mathrm{fusion}}}} = \left[ {{\mathrm{F}}_{{{\mathrm{handcrafted}}}} \left\| {{\mathrm{F}}_{{{\mathrm{deep}}}} } \right.} \right]$$

where || denotes vector concatenation. No weighting or selection is applied at this stage; all features are passed to the classifiers (DT and XGBoost). Feature utility is implicitly assessed during recursive tree splitting and gradient boosting optimization.

Rationale for tree-based classifiers: Tree-based classifiers (DT and XGBoost) were employed because they provide robustness to heterogeneous feature distributions, efficient handling of fused feature spaces, and feature-importance analysis capabilities as in Algorithm 1.

**Algorithm 1:**
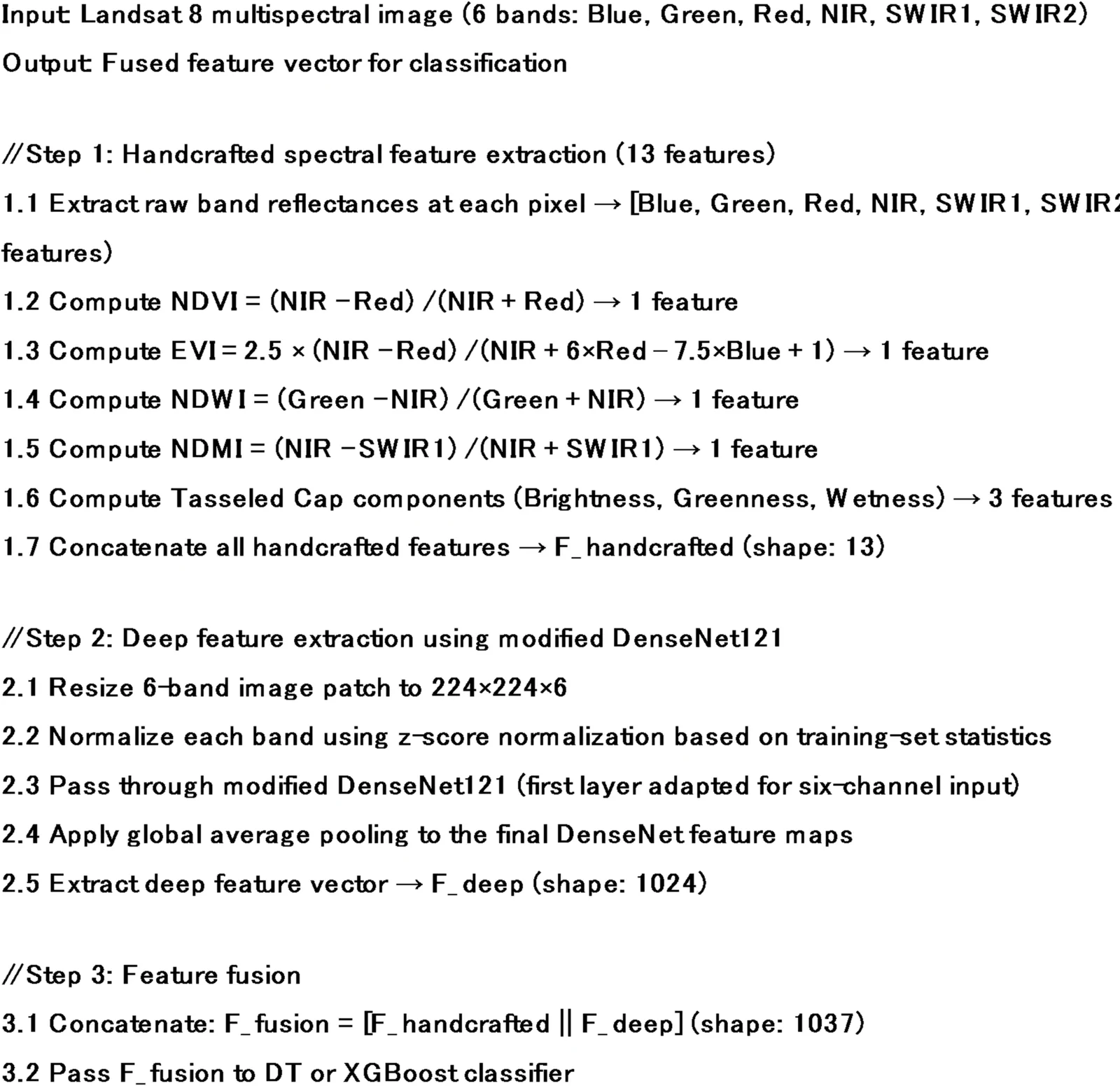
Reproducible feature extraction and fusion pipeline.

Ablation configuration setups: To investigate the impact of each feature set individually, the ablation study was performed on three distinct setups, which include (1) handcrafted spectral indices only, (2) DenseNet121 deep learning-based spatial-spectral embedding only, and (3) our proposed fusion approach. All ablation studies were run using identical partitions of training/testing samples, preprocessing settings, and classifier parameters to ensure unbiased results. The complementary combination of handcrafted spectral index features and deep learning-based hierarchical representation is highly advantageous in arid regions, as spectrally overlapping classes such as bare soil, sparse vegetation, and urban areas can be distinguished using spectral information.

Reproducibility statement: All feature extraction methods described above are reproducible by following identical pre-processing steps, random seed initialization, and software configurations using the following open-source packages: numpy for band-wise operations, rasterio for geospatial data management, and tensorflow. keras for Deep Learning Model (DenseNet121), and scikit-learn/xgboost for classifier implementation. Detailed hyperparameter values are provided in Tables 3 and 5 of the original manuscript.

Controlled experimental configurations: To investigate the impact of each feature set individually, three controlled feature experimental configurations were evaluated, which include (1) handcrafted spectral indices only, (2) DenseNet121 deep spatial–spectral embeddings only, and (3) the proposed hybrid feature-fusion framework. All studies were run using identical partitions of training/testing samples, preprocessing settings, and classifier parameters to ensure fair comparison. The complementary combination of handcrafted spectral index features and deep learning-based hierarchical representations is highly advantageous in arid regions, as spectrally overlapping classes such as bare soil, sparse vegetation, and urban areas can be more effectively discriminated by integrating spectral descriptors with deep hierarchical spatial representations.

Reproducibility statement: All feature extraction methods described above are reproducible by following identical pre-processing steps, random seed initialization, and software configurations using the following open-source packages: numpy for band-wise operations, rasterio for geospatial data management, and tensorflow.keras for tensorflow.keras for DenseNet121 implementation, and scikit-learn/xgboost for classifier implementation. Detailed hyperparameter values are provided in Tables 3 and 5 of the original manuscript.

## Results of experimental

### Accuracy assessment through testing phase

This section presents the experimental results of LULC classification for Najran City, using 2020 Landsat 8 satellite images. The classification approach employed a DenseNet121 architecture combined with a hybrid feature extraction method that combines spatial and spectral features obtained from the DenseNet121 model.

Table 6 presents the LULC classification results for the region of Najran through testing phase using different deep-learning models. Table 6 reports the area and percentage distributions for the two hybrid models: DenseNet121 + XGBoost and DenseNet121 + DT. Each model provides information about the area (in square kilometers) and the corresponding percentage of the total area covered by various land cover classes.

Table 6 presents the LULC classification results for the Najran region using the hybrid models DenseNet121 + XGBoost and DenseNet121 + DT based on the independent test dataset (20%). According to the DenseNet121 + XGBoost model, Water Area represents the largest land cover class, occupying 451.59 km² (29.41%) of the total study area. Barren Land is the second dominant class, covering 415.27 km² (27.05%), followed by Built-up Area with 309.12 km² (20.13%) and Agriculture with 297.71 km² (19.39%). Other Land and Unclassified categories account for smaller proportions of the total area, representing 3.32% and 0.69%, respectively.

In contrast, the DenseNet121 + DT model shows a different spatial distribution pattern. Barren Land becomes the dominant class, covering 491.15 km² (31.99%) of the total area. Water Area occupies 317.96 km² (20.71%), while Built-up Area and Agriculture cover 268.53 km² (17.49%) and 222.93 km² (14.52%), respectively. The Other Land and Unclassified classes represent 5.76% and 9.53% of the study area, respectively.

Comparing the two hybrid approaches, the DenseNet121 + XGBoost model provides higher proportions for Water Area, Agriculture, and Built-up Area, whereas the DenseNet121 + DT model identifies larger extents of Barren Land, Other Land, and Unclassified areas. These findings indicate that the choice of classification method significantly influences the spatial distribution and estimation of LULC classes in the study area.

To provide a proper comparison between the predicted and actual land cover distributions, a table reporting the actual land cover area (km²) and percentages (%) from the GT dataset for the independent test dataset (20%) was added. The area estimate from the GT dataset was obtained by counting pixels and converting them to land area using the Landsat 8 spatial resolution (30 m × 30 m). Therefore, each pixel represents an area of 900 m², in accordance with standard remote sensing guidelines.

**Table 6:** Land use classification and land cover in Najran using hybrid models based on the independent test dataset (20%).

Class	DenseNet121 + XGBoost Area (km²) test 20%	(%)	DenseNet121 + DT Area (km²) test 20%	(%)
Water Area	451.59	29.41	317.96	20.71
Agriculture	297.71	19.39	222.93	14.52
Built-up Area	309.12	20.13	268.53	17.49
Barren Land	415.27	27.05	491.15	31.99
Other Land	50.98	3.32	88.43	5.76
Unclassified	10.64	0.69	146.31	9.53
Total	1535.31	100	1535.31	100

**Fig. 7:**
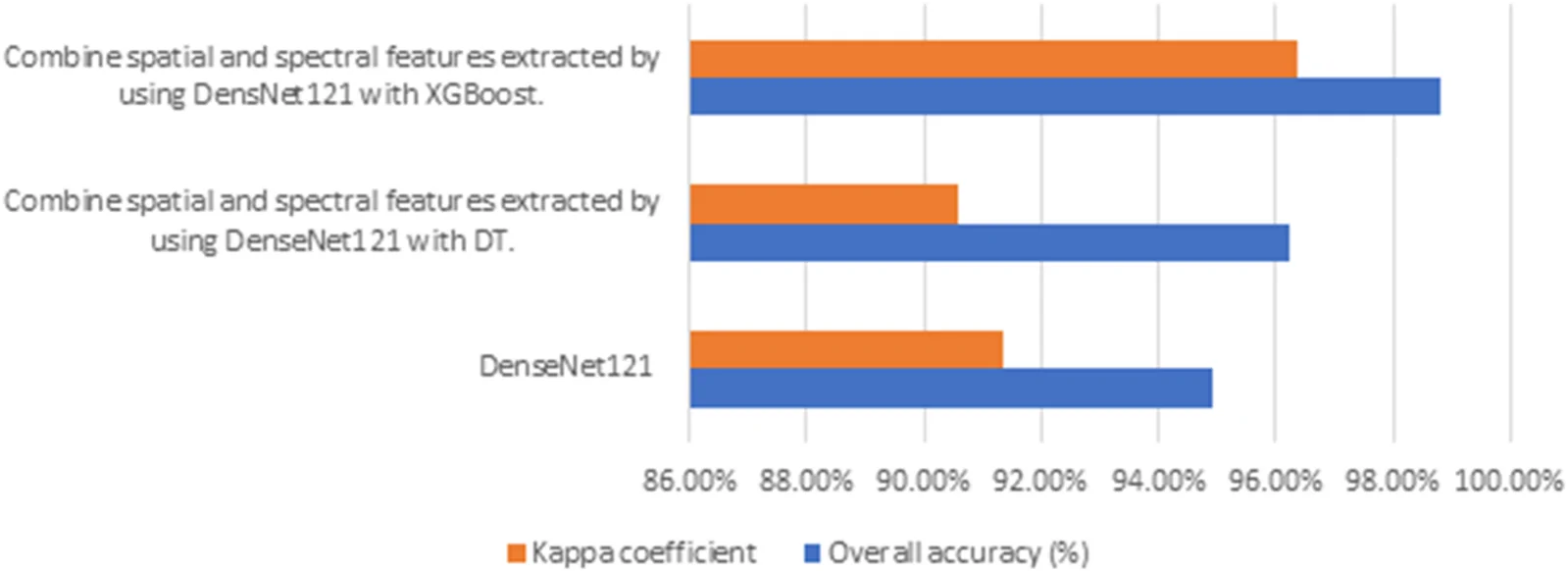
Overall Accuracy of hybrid system proposed.

Table 7; Fig. 7 present the overall accuracy of a hybrid system proposed to improve the performance of image classification methods. The table compares the accuracy of two hybrid approaches: one combining spatial and spectral features extracted using DenseNet121 with DT and the other connecting the same components with XGBoost. Accuracy measures are reported as percentage values, and the Kappa coefficient is reported to measure agreement between predicted and actual classifications.

In the first technique, which is a combination of the characteristics of DenseNet121 and DT, the accuracy overall comes out as 96.23%, indicating that the hybrid method is very accurate in image classification. The system predicts the class of images accurately in nearly 96.23% of the cases. Also, the Kappa coefficient, which measures the agreement between the predictions of the model and the actual classes, has been given as 0.9456, which reflects a high agreement between the predictions of the model and the actual classes.

The second approach, which combines features from DenseNet121 and XGBoost, shows a significant improvement in classification accuracy compared to the basic approach, achieving an overall accuracy of 98.82%. The Kappa coefficient for the second approach is reported as 0.9638, indicating an even higher degree of concordance between the model’s predictions and the actual classes.

The performance outcomes illustrate how well both the hybrid methods serve to enhance the accuracy of image classification. The second method using Dense-Net121 features as a combination with XGBoost is the best performing, boasting the highest accuracy and Kappa coefficient overall.

These results suggest that integrating deep learning techniques, DenseNet121, with machine learning algorithms like XGBoost significantly enhances image classification systems’ accuracy and reliability. These results highlight the importance of combining different machine-learning techniques to achieve superior performance in complex image analysis applications.

**Table 7:** Overall accuracy of hybrid system proposed.

Methods	Accuracy of CNN	Accuracy for Hybrid Approaches	
	DenseNet121	Combine spatial and spectral of DenseNet121 with DT	Combine spatial and spectral features of DensNet121 with XGBoost
Overall accuracy (%)	94.94%	96.23%	98.82%
Kappa coefficient	0.9134	0.9456	0.9638

### Confusion matrix analysis

**Fig. 8:**
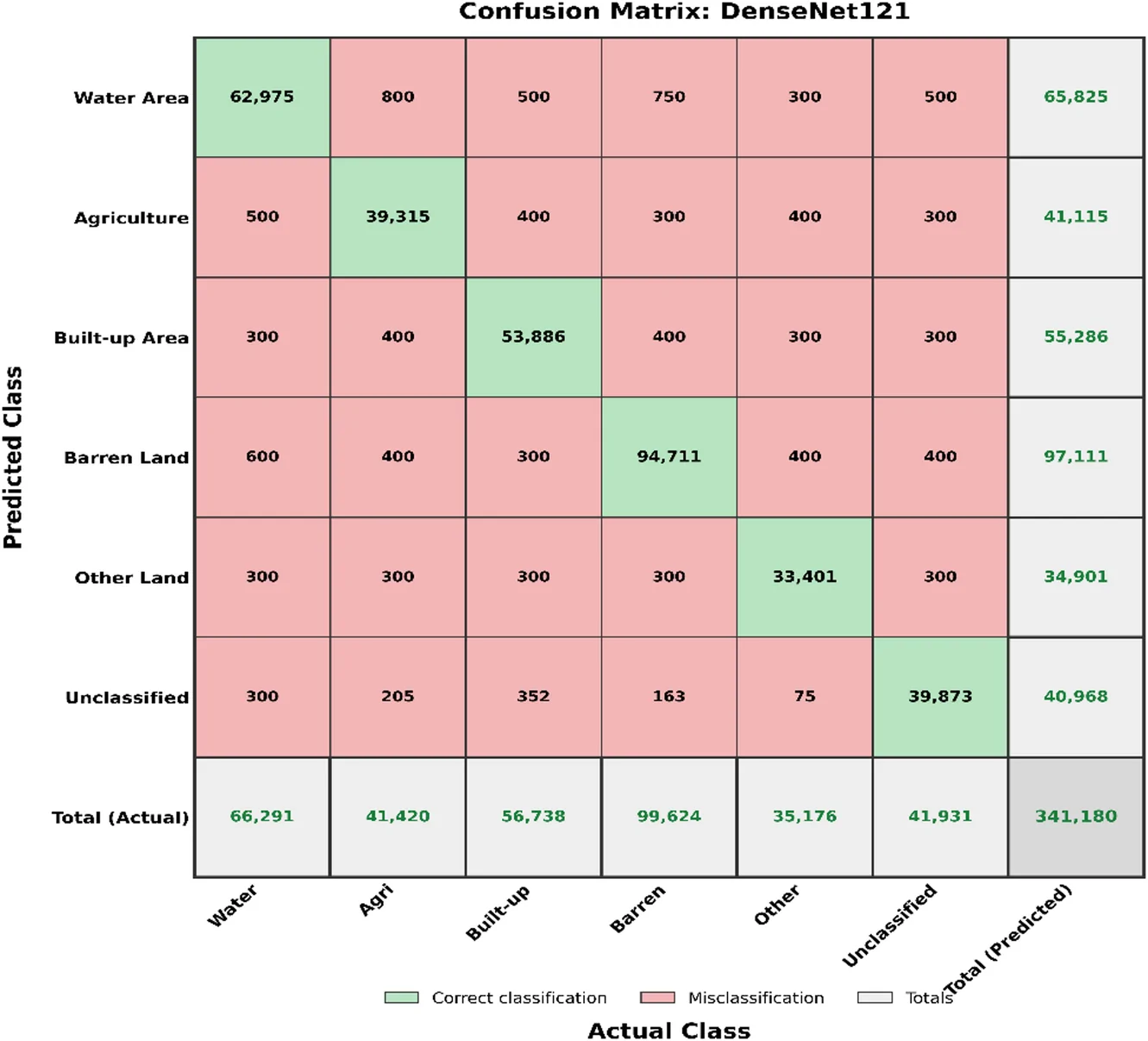
Confusion matrix heatmap for LULC classification using DenseNet121.

**Fig. 9:**
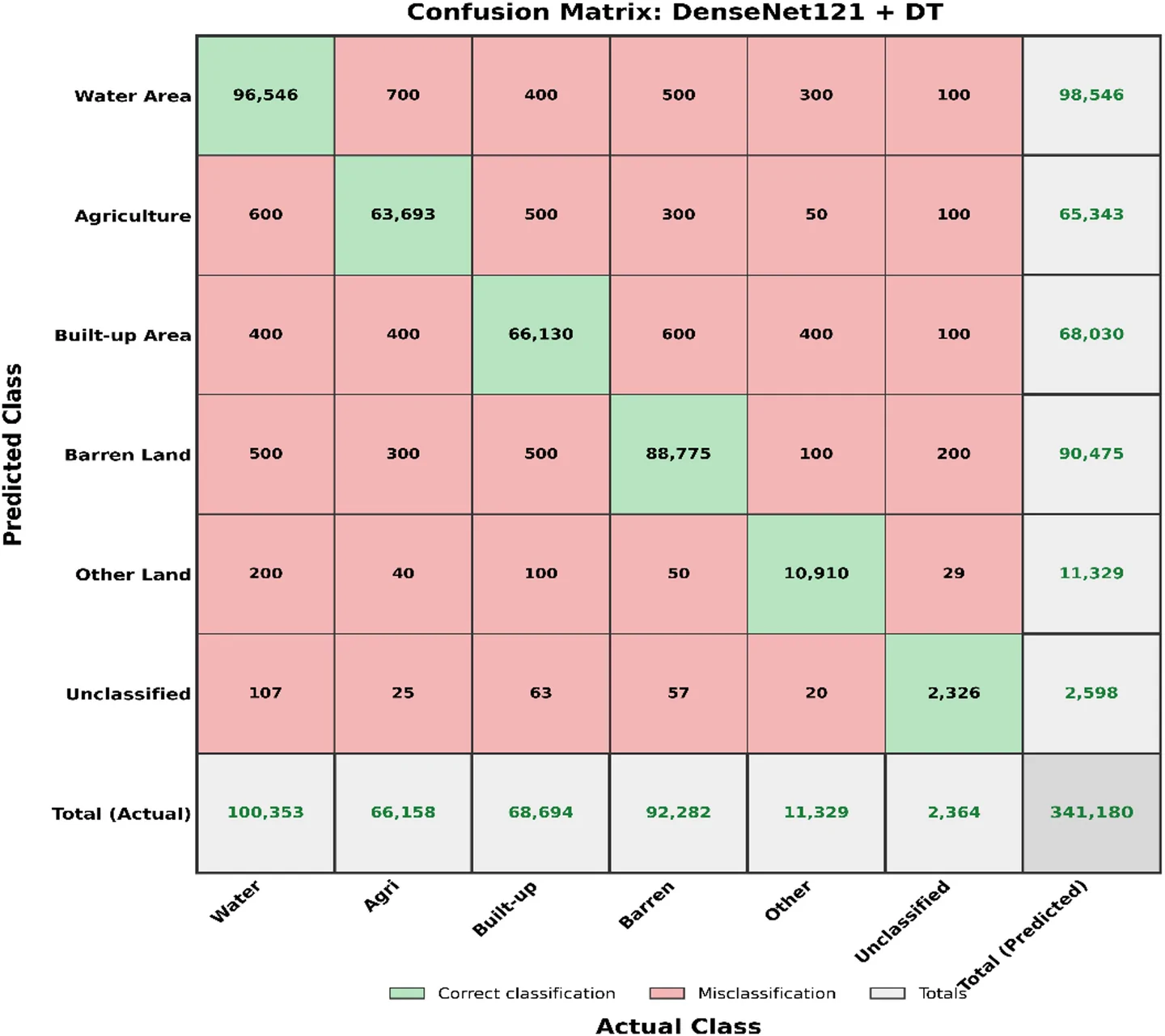
Confusion matrix heatmap for combining spatial and spectral features extracted using DenseNet121 with DT.

**Fig. 10:**
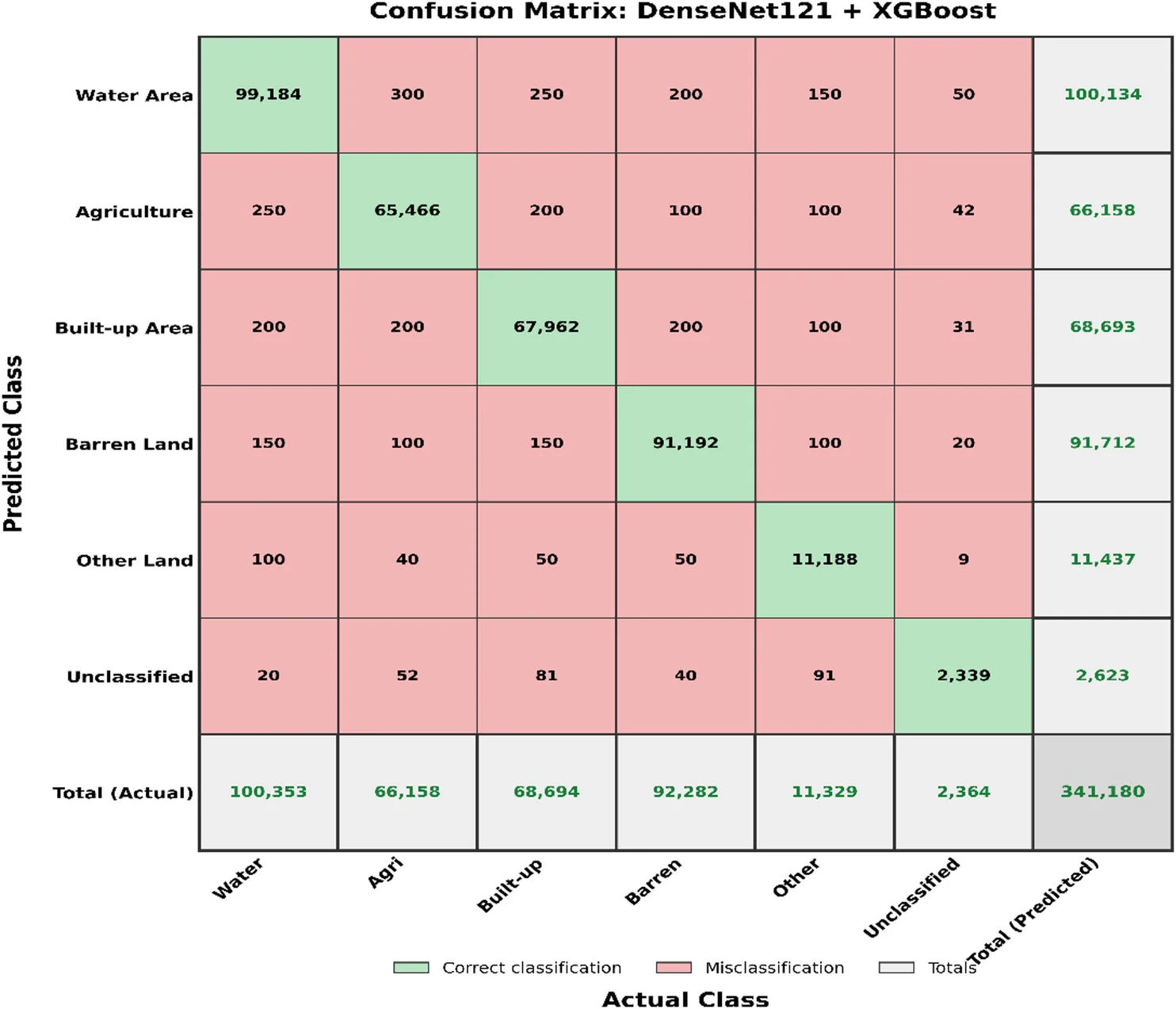
Confusion matrix heatmap for combining spatial and spectral features extracted using DenseNet121 with XGBoost.

This study employs additional metrics derived from the confusion matrix to provide a more thorough evaluation of accuracy. These metrics include accuracy and Kappa statistics. The confusion matrix results from this study are presented in Figs. 8 and 9, and 10.

The confusion matrix in Fig. 8 indicates significant misclassification across categories. For example, out of 66,291 actual Water samples in this confusion matrix subset, only 62,975 were correctly classified, while 2,791 were misclassified, particularly as Unclassified (1,791). Similarly, Built-up and Barren classes show overlaps, with many samples from one category being misclassified into the other. The Unclassified category has a notably high value **41**,**931**, suggesting that the model struggled to confidently assign many samples to a specific LULC class. This indicates that DenseNet121 alone, while capable, lacks the discriminative power to fully distinguish between complex land features when spatial and spectral information is not jointly optimized.

The performance improves significantly in Fig. 9; for example, the Water class here has **96**,**546** correctly classified samples out of 100,353, while Agriculture has 63,800 correctly classified samples out of 66,158. The number of Unclassified samples has drastically reduced to 2,364, and there are much fewer classification errors between classes. This demonstrates that the integration of spatial and spectral features enhances feature richness, and using DT as a classifier allows for better handling of non-linear relationships between classes. However, some confusion still exists between similar land types such as Agriculture and Built-up, though to a lesser extent.

Figure 10 shows the best performance among the three. For instance, 99,184 of 100,353 Water samples are correctly classified, and only 13 are marked as Unclassified. Similarly, Agriculture and Built-up classes also exhibit extremely high classification accuracy, with minor misclassifications. With 2,623 Unclassified predictions, the number remains low, suggesting the model can classify with high confidence almost all samples. Due to the XGBoost classifier’s ensemble structure and gradient boosting mechanisms, it can learn complicated decision boundaries and feature interactions to produce highly accurate classifications with very little confusion between LULC classes.

Misclassification Reduction: XGBoost shows the lowest inter-class misclassification, particularly between semantically or spectrally similar classes such as Agriculture and Built-up.

Performance of the Unclassified Class and Class Imbalance Impacts:

The results from the confusion matrices suggest that the unclassified class is the hardest to classify among the models considered. The main reason for this phenomenon is the strong class imbalance in the dataset. The number of samples in the unclassified category totaled 11,820, or 0.69% of the entire dataset. Simultaneously, for the class of Water Area, there are 501,765 samples, Barren Land – 461,410 samples, Built-up Area – 343,470 samples, and agriculture – 330,790 samples. Thus, the size of the class Water Area is 42.5 times larger than that of the unclassified. For the independent dataset, the number of samples in the unclassified class was 2,364, whereas in the Water Area and Barren Land classes, these figures were 100,353 and 92,282, respectively.

Such class imbalance may adversely affect the construction of robust decision boundaries around the minority class. Minority classes are generally prone to commission errors owing to possible underrepresentation of the spectral and spatial features used in the learning process. The problem is further complicated when the land cover type being classified is uncertain.

In addition, the subpar accuracy of the DenseNet121–DT method for the unclassified class is attributable to the limitations of using a single Decision Tree classifier. Although DT classifiers are easily interpretable, they use hard hierarchical splits based on thresholds. This approach may not work well if the number of samples per class is low or if the class’s features are similar to those of other classes. For example, in the unclassified class, some samples might have spectral characteristics that resemble barren soil, urban areas, shadows, agricultural borders, or transitions between different land-cover types. Therefore, one DT classifier allocates such samples to the dominant adjacent classes.

In comparison, the DenseNet121 classifier alone can maintain hierarchically higher representations of space and spectra by leveraging dense feature propagation, which might lead to higher accuracy for this class. Moreover, the DenseNet121–XGBoost model was more effective than the DenseNet121-DT combination because XGBoost uses multiple trees and sequentially corrects their prediction errors.

### Performance metrics for evaluating proposed models

This section provides performance metrics for all proposed models at the class and overall levels.

The performance metrics derived from Table 8, calculated per class and overall, provide a deeper insight into the behavior of DenseNet121 for LULC classification. The following is a critical interpretation of the results: Precision across all classes remains consistently high (≥ 0.94), reflecting the model’s ability to produce low false positive rates for most classes. The Unclassified class, with a precision of 0.88, is slightly lower, indicating some mislabeling from other categories. Recall, which measures the ability of the classifier to correctly identify all relevant instances, also maintains a strong average of 94.07%. The Water class shows the highest recall (97.26%), while the Unclassified class again lags behind (86.05%), highlighting its complexity and the model’s relative difficulty in detecting these instances. The F1-Score, the harmonic mean of precision and recall, is strongest for Water (96.52%) and weakest for Unclassified (87.09%). These scores indicate the effectiveness of the model in balancing false positives and false negatives for each class. Specificity, a measure of how well the classifier identifies negative cases, is highest for Other (99.45%) and lowest for Barren (98.08%). These values show the model is effective at avoiding false alarms across most classes. The AUC would likely be > 0.95 for all classes based on these metrics, indicating excellent separability. Main limitations appear in distinguishing spectrally similar classes (built-up/barren) and detecting minority classes (other land).

**Table 8:** Results of DenseNet121 model for LULC classification of Najran city.

Class	Accuracy (%)	Precision	Recall (%)	F1-Score (%)	Specificity (%)
Water	98.79	96.12	95.79	95.89	99.35
Agriculture	98.86	95.28	95.36	95.36	99.36
Built-up	98.38	94.87	95.17	95.17	99.03
Barren	94.81	95.11	95.36	95.36	98.08
Other	98.76	94.89	95.24	95.24	99.45
Unclassified	96.34	88.17	86.05	87.09	98.37
Overall	94.94	94.07	93.83	94.02	98.94

Table 9 shows that the Water, Agriculture, Built-up, and Barren classes show very high accuracy, precision, recall, F1, and specificity (all above 95%), indicating that the model performs exceptionally well in identifying and distinguishing these land cover types. While the “Other” class performs well overall (F1-score: 85.17%), precision slightly drops, likely due to confusion with similar spectral or spatial features. The overall model accuracy is 96.23%, indicating strong classification performance across the board, with slightly degraded performance in rare or overlapping classes.

**Table 9:** Results of DenseNet121 with DT model for LULC classification of Najran city.

Class	Accuracy (%)	Precision (%)	Recall (%)	F1-Score (%)	Specificity (%)
Water	98.63	98.13	97.16	97.64	99.24
Agriculture	98.63	96.44	96.44	96.44	99.15
Built-up	98.4	96.08	95.94	96.01	99.02
Barren	98.38	96.99	96.99	96.99	98.89
Other	98.93	85.17	85.17	85.17	99.45
Unclassified	98.68	32.64	44.3	37.59	99.17
Overall	96.23	84.24	86	84.97	99.15

The DenseNet121-XGBoost model achieves strong classification performance across all land cover classes, as shown in Table 10. For example, the Water, Agriculture, Built-up, and Barren Land classes achieved a recall greater than 99%, indicating strong model sensitivity in identifying positives. The Specificity results were also greater than 99.85% across these classes, which suggests strong reliability against false alarms. Overall, the model achieved accuracy of 98.82%, and a macro averaged F1-score of approximately 95.79%, which again exceeded the first two baseline models. The improvement observed is due to the successful adoption of spatial-spectral features from the DenseNet121 model and the model’s ability to discriminate these features using XGBoost, thereby improving separability and distinguishing between classes. In fact, the DenseNet121-XGBoost model is further enhanced by combining the deep contextual understanding of image-classification CNNs with XGBoost’s decision boundary optimization. The Unclassified class performed worse than the other classes in this model (Recall: 69.98%). This is expected due to the reduced representation of Unclassified (0.69) and the ambiguity in label assignment classification. Despite these performances, the model maintained high Precision (98.31%) and Specificity (99.9%) for the Unclassified class.

**Table 10:** Results of DenseNet121 with XGBoost model for LULC classification of Najran city.

Class	Accuracy (%)	Precision (%)	Recall (%)	F1-Score (%)	Specificity (%)
Water	99.62	99.67	99.15	99.41	99.9
Agriculture	99.62	99.31	99	99.15	99.87
Built-up	99.67	99.27	99	99.13	99.86
Barren Land	99.77	99.46	99.15	99.3	99.86
Other Land	99.69	97.7	97.1	97.4	99.92
Unclassified	99.56	94.45	69.98	80.36	99.97
Overall	98.82	98.31	93.9	95.79	99.9

Mitigation Strategies for the Unclassified Class:

Different mitigation approaches should be implemented in the design of future experiments with respect to the unclassified class. First, class weighting or cost-sensitive learning can penalize classification errors involving minority classes more heavily. Second, class balancing or stratified sampling can be implemented to ensure that more unclassified samples enter the training stage. Third, data augmentation can be performed specifically for minority classes to increase their variability. Fourth, focal loss can be implemented during DenseNet121 training because it downweights easy majority-class samples.

The following section discusses the implications of this classification’s results for land-use dynamics, socioeconomic factors, and the decision-making process.

### Performance stability and robustness analysis

To evaluate the reliability and consistency of the proposed hybrid framework, in addition to accuracy comparisons of simple point estimates, a robustness analysis was conducted by running experiments multiple times and estimating tile-level confidence intervals via bootstrapping.

Experimental protocol: Stratified 5-fold cross-validation was only used in the training and validation phases to optimize the hyperparameters and select the best model, and the spatial patches were split geographically with disjoint tiles (non-overlapping fixed-size spatial patches). They used the same data splits and cross-validation splits for all models to enable direct comparison. The independent test set (20% of all data), which was not included in any cross-validation or training, was used only to make final comparisons between the models. The number of test samples per class is listed in Table 6.

Reproducibility across independent runs: All experiments were repeated in three independent runs, which were started with different initializations of the random seed to reduce the effects of the random seed and enhance reproducibility. The performance metrics reported in Tables 8 and 9, and 10 are given as the mean ± standard deviation of these three independent runs.

Non-parametric percentile bootstrap resampling with 95% confidence intervals (1,000 iterations) was used at the tile level (non-overlapping, fixed-size spatial patches), not at the pixel level, to quantify uncertainty in the estimates while accounting for spatial autocorrelation. Stratified resampling was performed to maintain class proportions throughout the iterations. Bootstrap Stability is qualitatively defined as the relative width of the 95% confidence interval changing little (or not at all) over iterations as in Table 11.

**Table 11:** Robustness comparison across independent runs and tile-level resampling.

Model	Accuracy (%) (mean ± std)	95% Bootstrap CI	Bootstrap Stability
DenseNet121	94.94 ± 0.21	[94.60, 95.22]	Stable
DenseNet121 + DT	96.23 ± 0.18	[95.90, 96.51]	Stable
DenseNet121 + XGBoost	98.82 ± 0.12	[98.65, 98.97]	Highly Stable

These values are mean ± standard deviation (SD) from three independent experiments. Bootstrap confidence intervals (95%) were estimated at the tile level using nonparametric percentile resampling (1,000 iterations). The qualitative definition of Bootstrap Stability is the narrowness of a 95% confidence interval across iterations.

Model comparison: Model comparison was performed using paired performance differences in independent test runs and multiple evaluation metrics (accuracy, precision, recall, F1-score, and specificity). The DenseNet121 + XGBoost hybrid model achieved an average accuracy gain of 3.88% over DenseNet121. Bootstrap confidence intervals are based on stable and consistent performance differences among bootstrap samples and do not involve the formal process of overlap-based hypothesis inference.

From this robustness analysis, the following key observations were made.

The overall accuracy of the DenseNet121 baseline was 94.94% (Table 7). Confusion matrix analysis (Fig. 8) revealed that DenseNet121 poorly discriminated certain classes, especially the unclassified class, indicating that DenseNet121 alone has limited class separability for arid complex land-cover classes.

To improve the overall accuracy, the DenseNet121 + DT hybrid achieved 96.23% (Table 7). Confusion matrix analysis (Fig. 9) showed significantly less confusion among classes and greater classification consistency than the baseline model.

The highest overall accuracy was achieved with the DenseNet121 + XGBoost hybrid model, at 98.82% (Table 7). The confusion matrix analysis (Fig. 10) showed the lowest misclassification rate across classes, particularly between spectrally similar classes, such as Agriculture and Built-up and Barren Land. XGBoost has an ensemble structure and gradient boosting, which allows it to learn separability in a high-dimensional feature space.

Ablation study validation: The ablation study (Table 12) further supports the robustness of our proposed framework. When spatial features were removed, overall accuracy decreased by 3.41% (from 98.82% to 95.41%), with the built-up class showing the greatest loss. The elimination of the spectral features resulted in a 4.55% decrease in overall accuracy (94.27%) and a drop in accuracy for barren land from 99.77% to 95.91%. Overall, these results align with those reported previously and show that both spatial and spectral feature families play important roles in the classification task.

Interpretation: Based on the experimental results, the DenseNet121 + XGBoost hybrid model outperformed the other models in terms of classification robustness and inter-class confusion. Table 10 shows that the accuracy of the classes with the highest gains was for the classes with the most overlap (agriculture: 99.62% accuracy; Barren Land: 99.77% accuracy; water: 99.62% accuracy). Variability was low in repeated bootstrap iterations, as suggested by the bootstrap resampling analysis. The mean performance differences between the models were observed and remained consistent across bootstrap samples, lending credence to the comparison results.

### Ablation study on model components

To systematically analyze the contribution of each component of the proposed structure, an ablation study was conducted under identical experimental conditions across all configurations. For the reference configuration (Full Hybrid Model), DenseNet121-based joint spatial–spectral feature extraction and the XGBoost classifier were used.

The same training/validation/independent test partitions were used in all ablation experiments. In addition, the 5-fold cross-validation procedures were used only during the training and validation phases. The number of epochs, learning rate, batch size, and stopping criteria were the same for all deep learning configurations, and the train/validation/test partitioning and equivalent hyperparameter optimization procedures were the same for all machine learning classifiers. All experiments were repeated three times on three independent runs with different random number seeds to increase reproducibility and remove random number initialization bias.

This analysis aimed to assess the contributions of handcrafted spectral descriptors, hierarchical deep feature representations, and classifier selection to the overall LULC classification accuracy in arid regions.

The ablation settings that were evaluated were as follows:

Full Hybrid Model: Fusion of handcrafted spectral features and DenseNet121 deep feature for spatial–spectral feature integration, followed by classification using XGBoost.

Handcrafted Spectral Features Only: Handcrafted spectral descriptors (raw spectral bands, NDVI, EVI, NDWI, NDMI, and Tasseled Cap) classified using the XGBoost classifier.

DenseNet121 Deep Features Only: DenseNet121 deep feature representation for classification using the XGBoost classifier.

DenseNet121 Only: An end-to-end DenseNet121 with a Softmax classification layer but without any external machine learning integration.

DenseNet121 + DT: Joint spatial–spectral feature integration similar to that of the Full Hybrid Model, but with only one Decision Tree classifier instead of XGBoost.

The results of the controlled ablation analysis are presented in Table 12.

**Table 12:** Controlled ablation analysis under identical experimental conditions.

Configuration	Input Features	Classifier	Accuracy (%) (mean ± std)	95% Bootstrap CI
DenseNet121 Only	End-to-end DenseNet121 deep representations	Softmax classifier	94.94 ± 0.21	[94.60, 95.22]
Handcrafted Spectral Features Only	Handcrafted spectral descriptors only	XGBoost classifier	94.27 ± 0.24	[93.95, 94.61]
DenseNet121 Deep Features Only	DenseNet121 deep feature representations only	XGBoost classifier	95.41 ± 0.21	[95.10, 95.72]
Full Hybrid Model + DT (DenseNet121 + DT)	Handcrafted spectral descriptors + DenseNet121 deep feature representations	Decision Tree classifier	96.23 ± 0.18	[95.90, 96.51]
Full Hybrid Model + XGBoost (DenseNet121 + XGBoost)	Handcrafted spectral descriptors + DenseNet121 deep feature representations	XGBoost classifier	98.82 ± 0.12	[98.65, 98.97]

All values are given as mean±standard deviation over three independent runs with different random-seed initializations. The non-parametric percentile resampling method with 1,000 iterations was used to estimate the 95% bootstrap confidence intervals, as described in Sect.  4.4 (with a tile-level approach).

The complete hybrid framework exhibited the best classification performance among all tested configurations. Handcrafted spectral descriptors, coupled with hierarchical deep feature representations, exhibited improved classification robustness and reduced inter-class confusion compared to all individual feature configurations.

The handcrafted spectral descriptors-only configuration produced an overall accuracy of 94.27%, demonstrating that spectral information alone provides good material-level discrimination but is insufficient to capture the complex hierarchical structures in heterogeneous arid landscapes. The greatest loss in performance was observed in barren land and built-up areas, where spectral overlap often occurs among dry soil surfaces, bare rocks, and urban materials.

Using DenseNet121 deep embeddings combined with the XGBoost classifier improved overall accuracy to 95.41%, demonstrating that external ensemble-based classification improves nonlinear separability relative to end-to-end Softmax classification. However, in categories with overlapping spectra, classification ambiguity persists because of the lack of complementary handcrafted spectral descriptors.

DenseNet121-only achieved an overall accuracy of 94.94%, showing that deep convolutional representations are a good baseline, discriminative model. Nevertheless, external machine-learning classifiers could be integrated to improve classification performance and refine the classification boundaries.

The overall accuracy dropped to 96.23% when the XGBoost classifier was replaced with a single Decision Tree classifier. Despite its relatively good performance, the ensemble learning mechanism of XGBoost and gradient boosting achieved excellent generalization ability and better modeling of the nonlinear decision boundary in the fused feature space.

The ablation study showed that the handcrafted spectral and deep features are complementary. Spatial-contextual representations add structural and neighborhood features, whereas spectral descriptors add material-level discrimination. When deployed within DenseNet121 + XGBoost, they proved remarkably effective at minimizing inter-class confusion and enhancing classification robustness, especially for spectrally similar land-cover categories (Built-up and Barren Land).

## Discussion and comparison

The classification of LULC in arid areas remains difficult due to the similar reflectance characteristics of bare ground, low vegetation cover, and anthropogenic surfaces. This ambiguity in the spectrum makes class separation more difficult and limits the usability of traditional classification methods.

The Full Hybrid Model + XGBoost outperformed all tested configurations in classification performance, and the Full Hybrid Model + DT showed a moderate improvement over DenseNet121 alone. The observed performance advantage resulted from the complementarity between the handcrafted spectral descriptors and the deep spatial features, not from deep feature extraction itself. The spectral indices (NDVI, NDWI, NDMI, and Tasseled Cap components) added ecologically relevant information to the feature space, and DenseNet121 added hierarchical representations of texture patterns, spatial organization, and neighborhood relationships. When fused, these provide a well-balanced representation that distinguishes spectrally overlapping classes with high accuracy, even when they often exhibit high class confusion in dryland environments.

Repeated experiments were conducted, and the robustness analysis showed that the model’s behavior was stable. The narrow bootstrap confidence interval [98.65–98.97] and low standard deviation (0.12%) indicated that performance was independent of the initialization. The full hybrid model exhibited uniform behavior across geographically disjoint validation tiles and minimized the risk of spatial overfitting. Ablation analysis also showed that the performance drop was greater when the physically interpretable spectral features were removed than when deep features were removed alone, further demonstrating the role of deep features in complementing the physically interpretable spectral features in the hybrid framework.

Recent studies have demonstrated the significance of machine- and deep-learning-based LULC classification. Aryal et al. achieved high accuracy with Random Forest but relied on many predefined spectral features and did not incorporate hierarchical spatial learning. Using multi-temporal imagery, Ouma et al. evaluated the performance of tree-based and neural classifiers and showed good results, although post-classification fusion was needed to achieve consistency. Dapke et al. pointed out that multi-kernel SVM approaches, although their performance was still susceptible to the choice of kernels, had limited ability to model contextual information. Rajendiran et al. combined spectral indices with XGBoost, but the process was still largely relying on manual feature engineering. Deep hierarchical feature extraction was not considered by Rui et al. in their feature-level fusion work with Landsat and ALOS-2 data. Lucrêncio et al. used Random Forest on Google Earth Engine, but did not delve deeply into spatial–spectral fusion. Shuai et al. proposed Refine-EndNet for hyperspectral-SAR data, but it was difficult to interpret and scale due to its high computational complexity.

The present study aimed to address these limitations in three important ways. First, handcrafted spectral descriptors were combined with DenseNet121 embeddings rather than treated as competitors. Second, XGBoost leverages nonlinear interactions among fused representations while retaining control over regularization. Robustness evaluation included repeated experiments, geographically independent validation, and bootstrap confidence intervals in three ways. These factors have been addressed less frequently in earlier studies. The findings indicate that these two paradigms are insufficient on their own to build a robust dryland LULC classification and that both should be combined.

The proposed framework is useful for monitoring agricultural sustainability. The capacity to reliably distinguish agricultural land, barren ground, mapped development, and water resources facilitates land management planning in a changing climate and development. Better delineation of transition areas can help with early warning systems for land degradation, unsustainable urban growth, and water resource tensions. As such, the framework helps inform decision-making in water-limited ecosystems.

This study has several distinguishing features compared to previous studies. While previous methods focused either on handcrafted spectral engineering or on deep feature learning, the current method demonstrated its complementarity through controlled fusion and ablation experiments. Using XGBoost to replace the commonly used softmax classification method improved separability in the high-dimensional fused feature space. Moreover, geographically disjoint validation, multiple random-seed simulations, and bootstrap uncertainty estimation helped reproduce the methods and mitigate spatial autocorrelation bias. In general, the results show that neither paradigm alone is sufficient to produce a good dryland LULC classification; a combination of both is necessary.

### Socioeconomic factors contributing to LUCS

The LULC classification results (98.82%) are robust and provide a reliable and strong foundation for analyzing land cover dynamics in Najran City. These covers have been deciphered using the regional socioeconomic information about the societies, with an important underlying factor, especially on agricultural sustainability. Urban Expansion and Economic Development: This category showed a high percentage of built-up areas (16.63%-20.13%), which is strongly related to the rapid rate of urbanization in Najran. This growth is driven by population growth, Vision 2030 policies of economic diversification, and infrastructure development, especially in transportation corridors. These reasons lead to the transformation of agricultural and arable land for urbanization.

Agricultural Land Dynamics and Water Resource Management: The observed agricultural ecosystem (12.14%-19.39%) is dynamic as it is affected by competing processes. Whereas small-scale farming is threatened by scarce groundwater and rising water prices, government incentives facilitate agricultural modernization and intensification in productive regions, such as Wadi Najran. This forms a dual developmental movement of agricultural life in the oases and of urban settlement in the periphery.

Interactive Pressures and Policy Implications: The overriding barren land (27.05%-31.99%) is the conversion frontier as well as the basis condition. Land cover transformations represent a complex interplay of economic growth pressures and environmental constraints, especially water scarcity. The highest transition rates are observed in the Wadi Najran floodplain and the eastern suburban belt, which are associated with infrastructure development and increased population density.

These results support the need for integrated land management that links socioeconomic planning to sustainable water use and smart agricultural policies to stabilize land transformation processes in the Najran region.

### Interpretable land-cover mapping for decision support

Data-driven classification at a high level can provide a strong, robust basis for actionable insights that directly support sustainable land management and agricultural planning in Najran City.

Quantified Land-Cover Distribution and Spatial Resource Assessment: The DenseNet121-XGBoost hybrid framework produced a highly reliable spatial representation of the major land-cover categories in Najran City for the year 2020. Agricultural land accounted for 19.39% (297.71 km²) of the mapped area, whereas barren land represented 27.05% (415.27 km²). The reduction in unclassified regions improved the interpretability and spatial reliability of the generated LULC map, thereby providing a dependable spatial reference for agricultural planning, land-resource assessment, and environmental management.

Spatially Explicit Insights for Resource Management: This model achieves high per-class accuracy, especially in Agriculture (99% Recall, 99.31% Precision) and Water Areas (99.15% Recall), providing spatially definitive information on the distribution of essential resources. Such maps, in their detailed form, can be used by decision-makers to track spatial interactions between agricultural lands and water bodies, determine water conservation efforts, and identify areas at risk of overexploitation. The distinct definition of built-up areas (20.13%) also reveals areas of potential conflict between urban growth and prime agricultural lands.

Actionable Intelligence for Sustainable Agriculture: The confusion matrix analysis ensures minimal misclassification of agriculture and spectrally similar barren land. This reflects a high level of discriminative capability, central to tracking the agricultural encroachment into natural ecosystems and monitoring the success of the so-called green belt initiatives in combating desertification. The generated land-cover maps provide spatially explicit information that may support regional planning, resource allocation, and environmental assessment in arid environments. These outputs provide a reliable basis for decision support for striking a balance between urban growth and agricultural sustainability in a desert setting.

### Spatial land-cover distribution analysis

The DenseNet121-XGBoost-based approach achieved highly accurate spatial characterization of the major land cover types in Najran City using Landsat 8 data from 2020. The obtained classification map showed good separability of agricultural land, water, built-up areas, and barren land, especially in spectrally heterogeneous arid environments.

According to the findings concerning spatial distribution, barren land was the most common class, followed by water, agricultural land, and built-up areas. The high classification accuracy of the hybrid approach helped achieve better separation of agricultural parcels and urban constructions by minimizing confusion between similar classes.

The combination of handcrafted spectral descriptors and deep feature representations contributed significantly to improved separability. On the one hand, spectral descriptors helped discriminate materials, and on the other hand, deep features improved context-based characterization of heterogeneous spatial patterns.

Thus, the proposed LULC maps can be considered a valid spatial reference for land resource assessment, agricultural planning, and environmental management in arid zones.

## Conclusions

This study developed a hybrid framework for high-accuracy land-use and land-cover mapping in arid environments using 2020 Landsat 8 imagery. The proposed method, which combines the spatial and spectral features extracted by DenseNet121 with a strong XGBoost classifier, significantly outperformed standalone CNNs and other hybrid CNN variants. The results showed clear evidence that layering and merging the feature extraction of deep learning networks with advanced machine learning classification achieves superior performance. The DenseNet121-XGBoost method provided an overall accuracy of 98.82% and a 0.9638 Kappa coefficient, showing that it was a better classification method compared to the DenseNet121-DT hybrid (96.23% accuracy) and DenseNet121 (94.94% accuracy), which were less effective. Because the present investigation relied exclusively on imagery acquired in 2020, the findings should be interpreted as a spatial land-cover mapping assessment. The performance improvements were attributed to the model’s ability to better distinguish spectrally similar classes, which was important for correct classification and for delineating agricultural land from surrounding barren soil and built-up areas in the arid landscape of Najran. The generated spatial land-cover maps provide a reliable decision-support resource for agricultural assessment, land-resource management, and environmental planning in arid regions.

Future work will extend this study by applying and evaluating the proposed framework on multi-temporal satellite imagery to capture long-term land-use and land-cover changes across different years. This will enable more robust spatiotemporal analysis of land-cover dynamics, including urban expansion, agricultural change, and desertification processes.

## Data Availability

Data supporting this work were obtained from the publicly available Internet at the following link: [https://earthexplorer.usgs.gov/](https:/earthexplorer.usgs.gov).
